# Primary cilia mediate mitochondrial stress responses to promote dopamine neuron survival in a Parkinson’s disease model

**DOI:** 10.1038/s41419-019-2184-y

**Published:** 2019-12-16

**Authors:** Ji-Eun Bae, Gil Myung Kang, Se Hee Min, Doo Sin Jo, Yong-Keun Jung, Keetae Kim, Min-Seon Kim, Dong-Hyung Cho

**Affiliations:** 1 Brain Science and Engineering Institute, Kyungpook National University, Daegu, 41566 Korea; 2 Asan Institute for Life Sciences, Asan Medical Center, University of Ulsan College of Medicine, Seoul, 05505 Korea; 3 Divison of Endocrinology and Metabolism, Asan Medical Center, University of Ulsan College of Medicine, Seoul, 05505 Korea; 4 School of Life Sciences, Kyungpook National University, Daegu, 41566 Korea; 5 Global Research Laboratory, School of Biological Sciences, Seoul National University, Seoul, 08826 Korea; 6 Department of New Biology, DGIST, Daegu, 42988 Korea

**Keywords:** Mitochondria, Mitochondria, Autophagy, Autophagy

## Abstract

A primary cilium is an antenna-like structure on the cell surface that plays a crucial role in sensory perception and signal transduction. Mitochondria, the ‘powerhouse’ of the cell, control cell survival, and death. The cellular ability to remove dysfunctional mitochondria through mitophagy is important for cell survival. We show here that mitochondrial stress, caused by respiratory complex inhibitors and excessive fission, robustly stimulates ciliogenesis in different types of cells including neuronal cells. Mitochondrial stress-induced ciliogenesis is mediated by mitochondrial reactive oxygen species generation, subsequent activation of AMP-activated protein kinase and autophagy. Conversely, abrogation of ciliogenesis compromises mitochondrial stress-induced autophagy, leading to enhanced cell death. In mice, treatment with mitochondrial toxin, MPTP elicits ciliary elongation and autophagy in the substantia nigra dopamine neurons. Blockade of cilia formation in these neurons attenuates MPTP-induced autophagy but facilitates dopamine neuronal loss and motor disability. Our findings demonstrate the important role of primary cilia in cellular pro-survival responses during mitochondrial stress.

## Introduction

An evolutionally-conserved organelle, the primary cilium, was once thought to be non-functional but is now considered a pivotal signaling center, associated with 15 signaling pathways including Hedgehog (Hh)^1,2^. During the assembly stage of the primary cilium, cilia-targeting proteins are transported from the Golgi to the basal body via vesicular transport, and then to the ciliary tip along the axoneme via intraflagellar transport (IFT)^1^.

Mitochondria, essential organelles for both cell survival and death continuously undergo balanced fission and fusion processes, which are termed mitochondrial dynamics^3^. Mitochondrial dynamics highly affect mitochondrial functions as well as their morphology. Thus, abnormalities in mitochondrial dynamics are directly linked to many human diseases, including neurodegenerative diseases^4^. Several GTPase proteins including dynamin-related protein 1 (Drp1), optic dominant atrophy 1 (OPA1), and mitofusin 1/-2 (Mfn1/2) regulate these dynamics^3^.

Autophagy is a catabolic process that degrades organelles and long-lived proteins and maintains cellular homeostasis by regulating the cellular energy balance and facilitating organelle quality control^5^. The autophagic machinery is highly sensitive to intracellular and extracellular stress cues^5^. The adaptive autophagy mechanism is a part of an integrated series of responses by which cells respond to stress stimuli^5^. Recent studies have elucidated a close reciprocal relationship between primary cilia and the autophagic machinery. Autophagy-related proteins (ATGs) shuttle to the basal body and primary cilia via IFT and Hh-dependent mechanisms^6^. The disruption of ciliogenesis and Hh signaling represses the autophagic capacity of serum-deprived cells^6^. Conversely, autophagy inhibits or simulates ciliogenesis through the elimination of IFT20 or oral-facial-digital syndrome 1 (OFD1), respectively^6,7^.

Growing evidence indicates an interplay between primary cilia and autophagy as well as between autophagy and mitochondrial dynamics/functions^8–11^. However, the evidence for an interplay between primary cilia and mitochondria is currently lacking. In the present study, we present new findings demonstrating that the primary cilium plays an important role in the integration of cellular responses to mitochondrial stress.

## Results

### Mitochondrial respiratory inhibitors and mitochondrial fission stimulate ciliogenesis

**Fig. 1:**
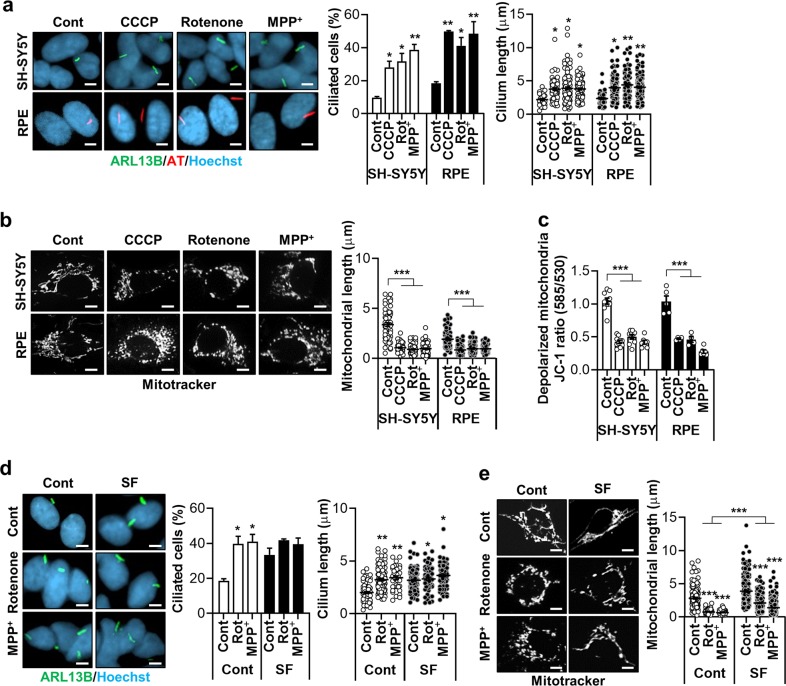
Mitochondrial respiratory inhibitors stimulate ciliogenesis. **a** Increased ciliogenesis occurs following treatment with the mitochondrial respiratory inhibitors CCCP (5 μM), rotenone (200 nM), and MPP^+^ (5 mM) in (upper) SH-SY5Y and (lower) RPE cells. Cells were cultured to almost 100% confluency and treated with these agents for 24 h. Primary cilia were stained with antibodies against ARL13B (green) or acetylated α-tubulin (AT) (red) and the nucleus (blue) was counterstained with Hoechst 33342 dye. **b** CCCP (5 μM), rotenone (200 nM), and MPP^+^ (5 mM) were applied to (upper) SH-SY5Y and (lower) RPE cells and mitochondria were stained with a MitoTracker (white) to measure mitochondrial length. **c** SH-SY5Y and RPE cells were treated with CCCP (5 μM), rotenone (200 nM), and MPP^+^ (5 mM). After 24 h, the alteration of mitochondrial membrane potential was measured with the MitoProbe JC-1 assay using the Attune NxT flow cytometer. **d**, **e** SH-SY5Y cells were treated with rotenone or MPP^+^ in the presence or absence of serum [normal (Cont) or serum-free (SF)]. Afterward, the ciliated cells and mitochondrial length were determined. Data are the mean ± SEM. *p < 0.05, **p < 0.01, ***p < 0.005 vs. untreated controls determined by ANOVA followed by a post hoc LSD test. Scale bar, 5 μm.

We screened for chemicals that regulate ciliogenesis using SH-SY5Y human neuroblastoma cells, and human retina pigment epithelial (RPE) cells. Primary cilia were monitored by staining with antibodies against ARL13B, a ciliary membrane protein with GTPase activity, or acetylated α-tubulin, a component of the ciliary axoneme. We observed that treatment with the mitochondrial respiratory complex-1 inhibitors rotenone and 1-methyl-4-phenylpyridinium (MPP^+^), strongly stimulated ciliogenesis in both SH-SY5Y and RPE cells, evidenced by an increase in the ciliary lengths and prevalence (Fig. 1a). Similarly, the frequency of cells with cilia and the average ciliary length more than doubled upon treatment with carbonyl cyanide m-chlorophenyl hydrazine (CCCP), a chemical uncoupler that collapses the mitochondrial membrane potential (Fig. 1a). In accordance with the previous notion, we found that treatment with rotenone, MPP^+^, or CCCP induced massive mitochondrial fragmentation and depolarization of the mitochondrial membrane potential (Fig. 1b, c).

Serum deprivation potently induces ciliogenesis by increasing the proportion of cells in Go phase and removal of secreted factors that inhibits ciliogenesis^12,13^. In addition, mitochondria fuse into a highly connected network during starvation^14^. We therefore tested whether mitochondrial respiratory inhibitor-driven ciliogenesis also occurs under serum-deprived conditions. SH-SY5Y cells were cultured with or without serum supplement during exposure to rotenone or MPP^+^ for 24 h. As expected, 24 h-serum removal increased both the ciliated cell frequency and the average ciliary lengths in these cells, whereas, treatment with rotenone or MPP^+^ did not induce additional cilium elongation in the serum-starved cells (Fig. 1d). Serum deprivation also slightly blocked the rotenone or MPP^+^-induced mitochondrial fission (Fig. 1e). Collectively, these data suggest that mitochondrial stress promotes ciliary growth.

**Fig. 2:**
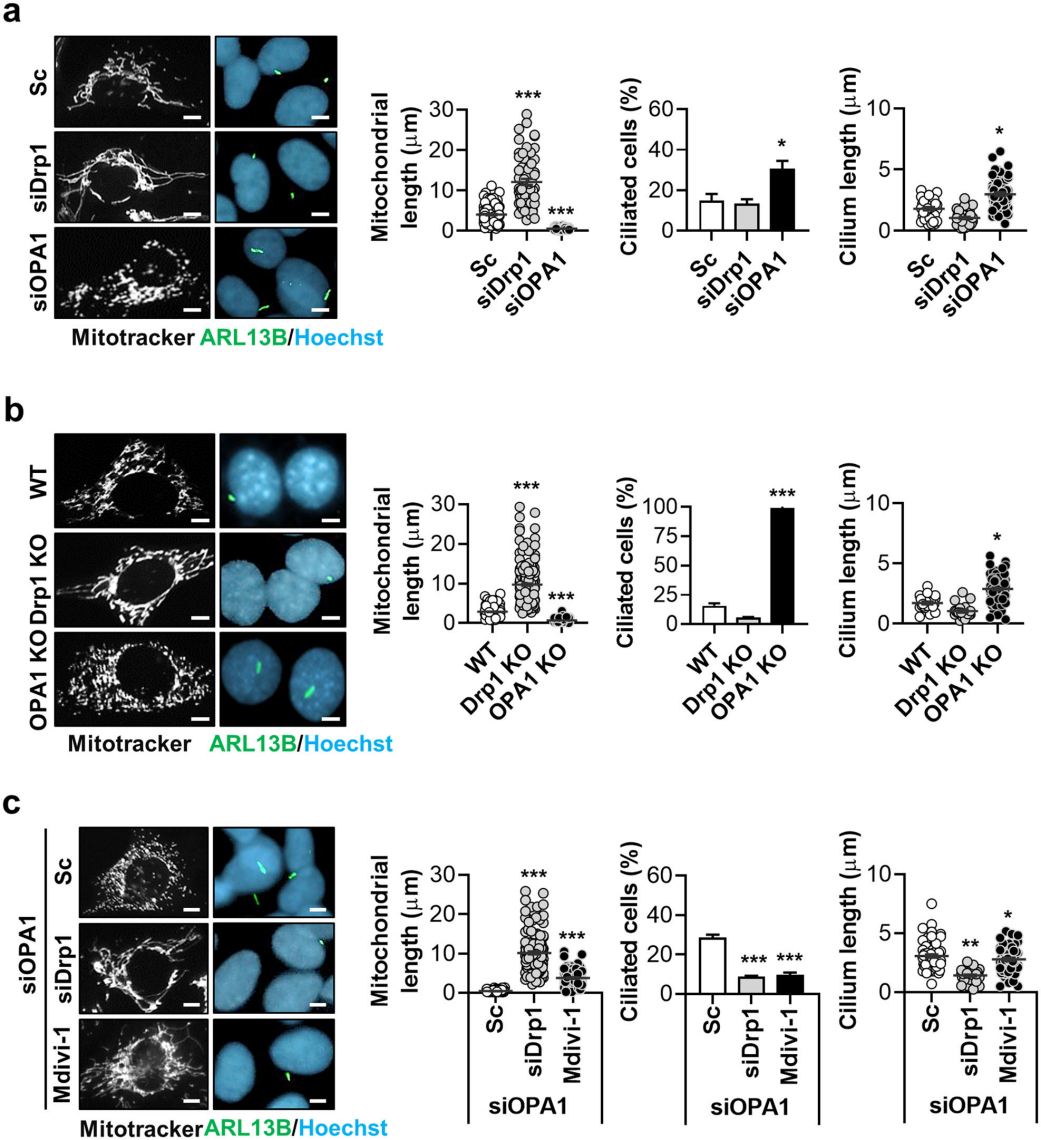
Mitochondrial fission induces ciliogenesis. **a** Effects of mitochondrial fusion induced by *Drp1* siRNA (si*Drp1*) treatment and fission by *OPA1* siRNA (si*OPA1*) treatment. SH-SY5Y cells transfected with si*Drp1* or si*OPA1* were stained with a MitoTracker (white), ARL13B (green), and Hoechst 33342 dye (blue). **b** *OPA1*-knockout (KO) mouse embryonic fibroblasts (MEFs) and *Drp1*-KO MEFs were stained with a MitoTracker (white), ARL13B (green), and Hoechst 33342 dye (blue). **c** Effect of mitochondrial fusion on ciliogenesis in SH-SY5Y cells with si*OPA1*-enhanced ciliogenesis. Mitochondrial fusion was induced by si*Drp1* transfection or Mdivi-1 (10 μM). Representative cilia images are presented. Cilia measurement data were obtained from about 200 cells per group and the experiments were repeated at least three times. Data are the mean ± SEM. *p < 0.05, **p < 0.01, ***p < 0.005 vs. scrambled non-targeting siRNA (Sc) treatment or wild type (WT) MEF cells determined by ANOVA followed by a post hoc LSD test. Scale bar, 5 μm.

Next, we investigated a possible link between mitochondrial fission/fusion and ciliogenesis. Mitochondrial dynamics are regulated by three GTPase family proteins. Drp1 triggers mitochondrial fission whereas OPA1 and Mfn1/2 mediate mitochondrial fusion^15^. We induced mitochondrial fusion using small inhibitory RNA (siRNA)-mediated Drp1 depletion and induced mitochondrial fission by *OPA1* siRNA treatment in SH-SY5Y and RPE cells. Successful induction of mitochondrial fusion and fission was confirmed by examining the mitochondrial morphology using Mito Tracker staining and the expression of OPA1 and Drp1 (Fig. 2a and Supplementary Fig. 1). Mitochondrial fission via OPA1 depletion robustly increased ciliogenesis, whereas mitochondrial fusion following Drp1 depletion had a minimal effect on ciliary frequency and lengths in either cell type (Fig. 2a and Supplementary Fig. 2). We further evaluated changes in the primary cilia of *Drp1*- and *OPA1*-knockout (KO) mouse embryonic fibroblast (MEF) cells. Nearly all of the *OPA1*-deficient MEF cells were ciliated, in contrast to ~20% of wild-type MEF cells (Fig. 2b). In addition, the cilia were significantly longer in the *OPA1*-KO MEF cells while, *Drp1*-KO MEF cells showed a mild decrease in the percentage of ciliated cells with no change in cilia length (Fig. 2b). However, in the serum-deprived condition, cilium length was not significantly elongated in *OPA1*-KO MEF cells (Supplementary Fig. 3). As <20% of the control siRNA-treated cells developed cilia, a reduction in ciliogenesis caused by mitochondrial fusion would likely be difficult to detect in *Drp1*-KO MEF cells. We thus examined the effect of mitochondrial fusion on cilia in SH-SY5Y cells with enhanced ciliogenesis mediated by OPA1 depletion. In these cells, the induction of mitochondrial fusion using *Drp1* siRNA and the mitochondrial fission inhibitor Mdivi-1 significantly suppressed ciliogenesis as well as mitochondrial fragmentation (Fig. 2c). These data indicate that mitochondrial fusion and fission control primary ciliogenesis in opposite directions.

### Mitochondrial ROS and AMPK mediate mitochondrial stress-induced ciliogenesis

**Fig. 3:**
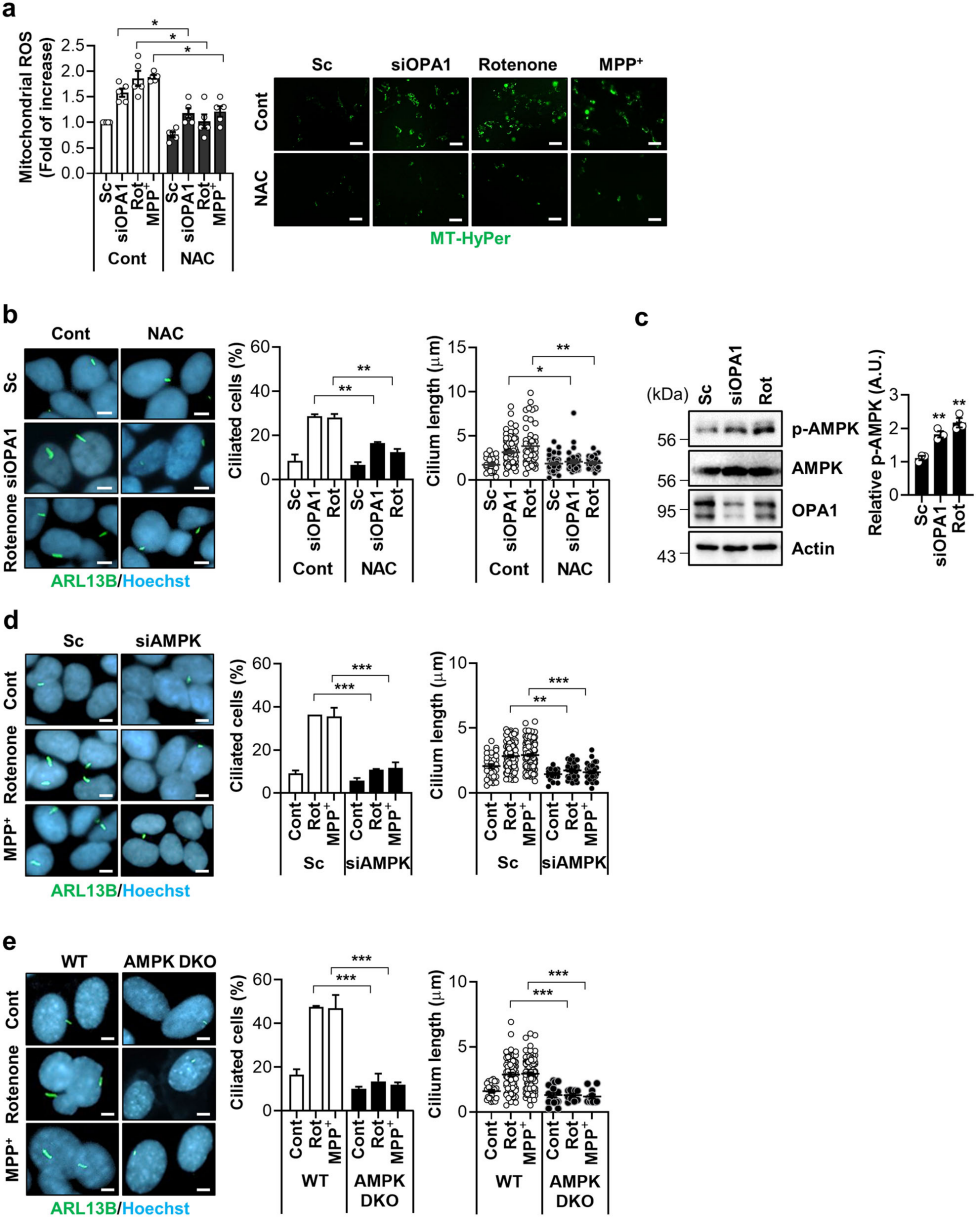
Mitochondrial ROS and AMPK mediate mitochondrial stress-induced ciliogenesis. **a**, **b** SH-SY5Y cells were transfected with scrambled control siRNA (Sc) or siRNA against *OPA1* (si*OPA1*). After 2 days the cells were treated with NAC (1 mM) for 24 h. SH-SY5Y cells were treated with rotenone (200 nM) or MPP^+^ (5 mM) with or without NAC (1 mM) for 24 h. The enhanced mitochondrial ROS (mtROS) formation. The level of mitochondrial H_2_O_2_ was measured using the fluorescent intensity of the Mito-HyPer. Scale bar, 20 μm. **b** NAC (1 mM) treatment blocks the induction of ciliogenesis by si*OPA1* or rotenone in SH-SY5Y cells. Primary cilia were immunostained with ARL13B antibody (green) and the nucleus was counterstained with Hoechst 33342 dye (blue). **c** SH-SY5Y cells transfected with si*OPA1* for 3 days or treated with rotenone (200 nM) were analyzed by Western blotting with a phosphorylated-AMPK (p-AMPK) (T172) antibody. **d** SH-SY5Y cells transfected with Sc or siRNA for *AMPK* (si*AMPK*) were further tread with rotenone (200 nM) or MPP^+^ (5 mM). After 24 h, the cells were stained with ARL13B (green) or Hoechst 33342 dye (blue). Scale bar, 5 μm. **e** *AMPK α1/α2* double-knockout (*AMPK* DKO) MEFs were treated with rotenone or MPP^+^. After 24 h, the cells were stained with ARL13B (green), Hoechst 33342 dye (blue). Scale bar, 5 μm. Experiments were repeated at least three times. Data are the mean ± SEM of about 200 cells per group. *p < 0.05, **p < 0.01, ***p < 0.005 between the indicated groups determined by ANOVA followed by a post hoc LSD test.

We next investigated the molecular mechanisms underlying mitochondrial stress-promoted ciliogenesis. Dysfunctional and fragmented mitochondria generate higher levels of reactive oxygen species (ROS). Mitochondrial-derived ROS (mtROS) act as a signal for various biological pathways^16^. To examine the role of mtROS in our system, cells were co-treated with an ROS scavenger, N-acetyl cysteine (NAC) in parallel with rotenone and *OPA1* siRNA treatment. The measurement of mtROS via the expression of a mitochondrial hydrogen peroxide sensor (MT-HyPer) revealed that NAC completely blocks mtROS overproduction caused by rotenone, MPP^+^, and the knockdown of OPA1 (Fig. 3a). A striking loss of rotenone- and OPA1 knockdown-induced ciliogenesis was observed in NAC-treated SH-SY5Y and RPE cells (Fig. 3b and Supplementary Fig. 4). NAC treatment also suppressed mitochondrial fission induced by OPA1 knockdown or rotenone in SH-SY5Y cells (Supplementary Fig. 5). These results suggest that mtROS critically mediates mitochondrial stress-induced ciliogenesis.

Adenosine monophosphate-activated protein kinase (AMPK) is a redox-sensitive cellular energy sensor activated by mtROS as well as ATP depletion^17^. AMPK acts to resolve low cellular energy and also oxidative stress^17^. Mitochondrial fission induced by *OPA1* siRNA or rotenone treatment increased AMPK α-subunit (T172) phosphorylation, a marker of AMPK activation, in SH-SY5Y cells (Fig. 3c). We thus tested whether AMPK is an important downstream mediator of mitochondria stress-related ciliogenesis. The downregulation of AMPK completely inhibited rotenone- or MPP^+^-induced ciliary changes in SH-SY5Y cells (Fig. 3d and Supplementary Fig. 6). Consistently, *AMPK-α1/α2* double-knockout (DKO) MEF cells failed to increase cilia formation or growth in response to OPA1 depletion, rotenone, or MPP^+^ treatment (Fig. 3e and Supplementary Fig. 7). However, treatment with rotenone or MPP^+^ increased mitochondrial fission in AMPK DKO MEF cells (Supplementary Fig. 7d). These data strongly indicate that AMPK activation is a critical event that connects mitochondrial stress to ciliogenesis.

### Inhibition of autophagy blocks mitochondrial stress-mediated ciliogenesis

**Fig. 4:**
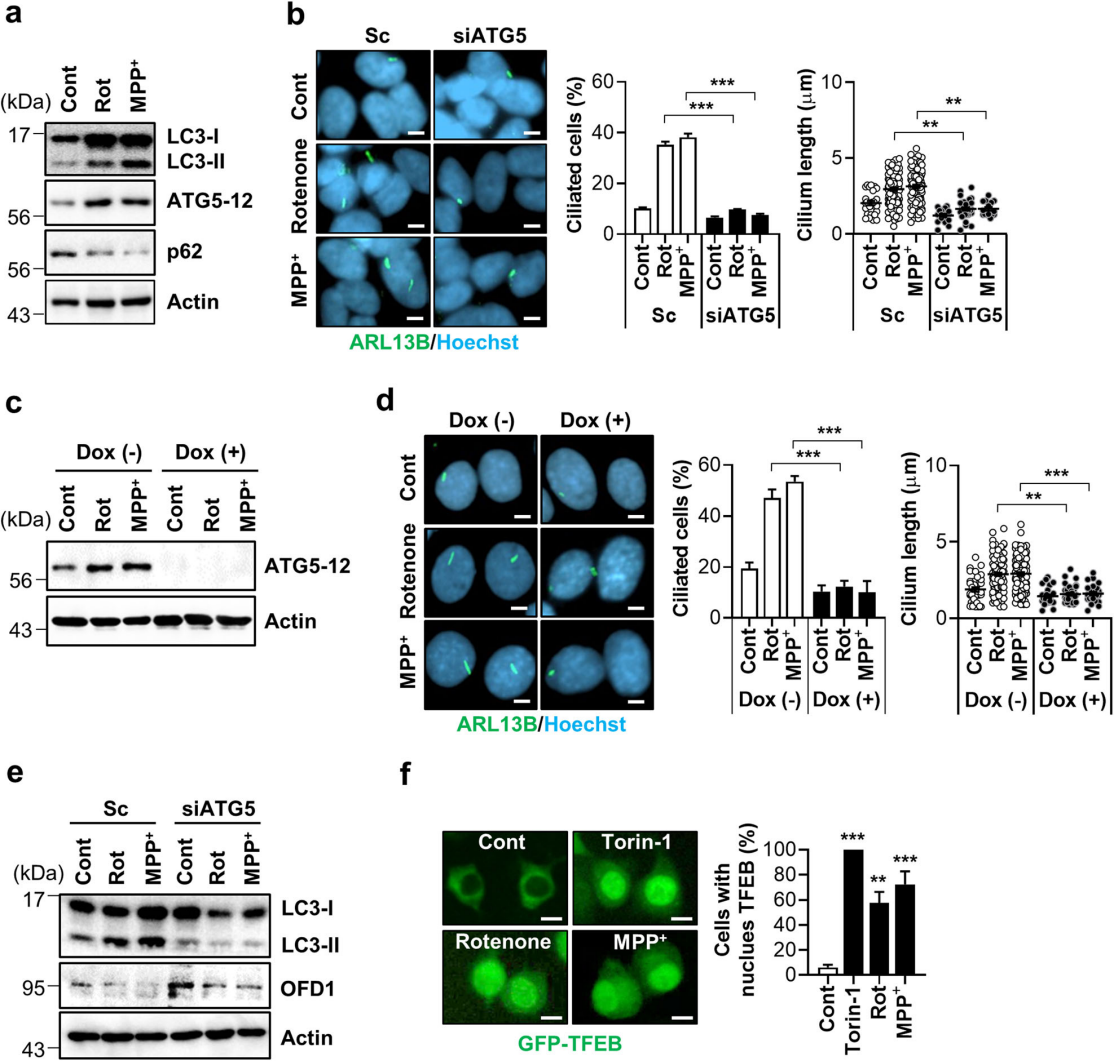
Inhibition of autophagy blocks mitochondrial stress-mediated ciliogenesis. **a** Rotenone and MPP^+^ treatment enhance autophagy in SH-SY5Y cells. SH-SY5Y cells were treated with rotenone (200 nM) and MPP^+^ (5 mM) for 24 h, then the cells were assessed by Western blotting with indicated antibodies. **b** SH-SY5Y cells transfected with si*ATG5* were further treated with rotenone (200 nM) and MPP^+^ (5 mM) for 24 h, then primary cilia were immunostained with ARL13B antibody (green) and the nucleus was counterstained with Hoechst 33342 dye (blue). Scale bar, 5 μm. **c**, **d** Blunted rotenone- and MPP^+^-driven ciliogenesis in M5-7 cells. MEFs under doxycycline (Dox)-induced ATG5 depletion (M5-7 cells) were treated to rotenone (200 nM) and MPP^+^ (5 mM) for 24 h with or without Dox. Dox-induced ATG5 depletion was confirmed by Western blotting with ATG5 antibody (**c**). **d** Primary cilia were immunostained with ARL13B antibody (green) and the nucleus was counterstained with Hoechst 33342 dye (blue). Scale bar, 5 μm. **e** SH-SY5Y cells transfected with si*ATG5* were further treated with rotenone (200 nM) and MPP^+^ (5 mM) for 24 h, then the cells were analyzed by western blotting with indicated antibodies. **f** SY5Y/GFP-TFEB cells were treated with Torin-1 (1 μM for 1 h), rotenone (200 nM, 24 h) and MPP^+^ (5 mM, 24 h). Then, nuclear localization of GFP-TFEB was observed. Scale bar, 1 μm. Data were obtained from about 200 cells per group and experiments were repeated at least three times. Data are the mean ± SEM. **p < 0.01, ***p < 0.005 between indicated groups determined by ANOVA followed by a post hoc LSD test.

AMPK activation increases autophagic flux through the inhibition of the mammalian target of rapamycin (mTOR) or direct activation of the mammalian autophagy-initiating kinase ULK1^18,19^. Autophagy is also triggered by mtROS via activated ataxia telangiectasia mutated (ATM)-liver kinase B1 (LKB)-AMPK signaling^20^. Moreover, autophagy has been suggested to be an important mechanism in serum starvation-induced ciliogenesis^7^. We therefore examined whether autophagy underlies the cellular ciliogenic responses to rotenone and MPP^+^ treatment. The results showed that rotenone and MPP^+^ treatment led to increased level of ATG5-12 conjugates and LC3-II accumulation (Fig. 4a and Supplementary Fig. 8), both indicative of enhanced autophagy. Notably, the inhibition of autophagy by ATG5 depletion completely prevented ciliary elongation elicited by rotenone and MPP^+^ treatment (Fig. 4b). Next, we additionally confirmed the effects in a doxycycline-induced *ATG5* knockdown cells. Likewise, MEF cells with a doxycycline-induced *ATG5* deficiency were unable to upregulate ciliogenesis in response to rotenone or MPP^+^ treatment but increased mitochondrial fission (Fig. 4c and Supplementary Fig. 9).

Degradation of OFD1 from the centriolar satellites has been suggested as a mechanism for inducible ciliogenesis in autophagy-induced cells^7,21^. Consistent with this notion, we observed a reduction in OFD1 expression upon rotenone and MPP^+^ treatment, which was attenuated in *ATG5*-deficient cells (Fig. 4e and Supplementary Fig. 10). These data further suggest that autophagy-mediated OFD1 degradation may contribute to mitochondria stress-driven ciliogenesis. Transcription factor EB (TFEB) is a master transcriptional regulator of lysosomal biogenesis and autophagy activation^22^. The nuclear localization of GFP-TFEB by TFEB activation was remarkably enhanced in response to treatment with rotenone, MPP^+^, and the mTOR inhibitor Torin-1 (Fig. 4f). Treatment with rotenone and MPP^+^ induced mitochondrial fission in Torin-1 treated cells (Supplementary Fig. 11).

### Ciliogenesis promotes cell survival under mitochondrial stress

**Fig. 5:**
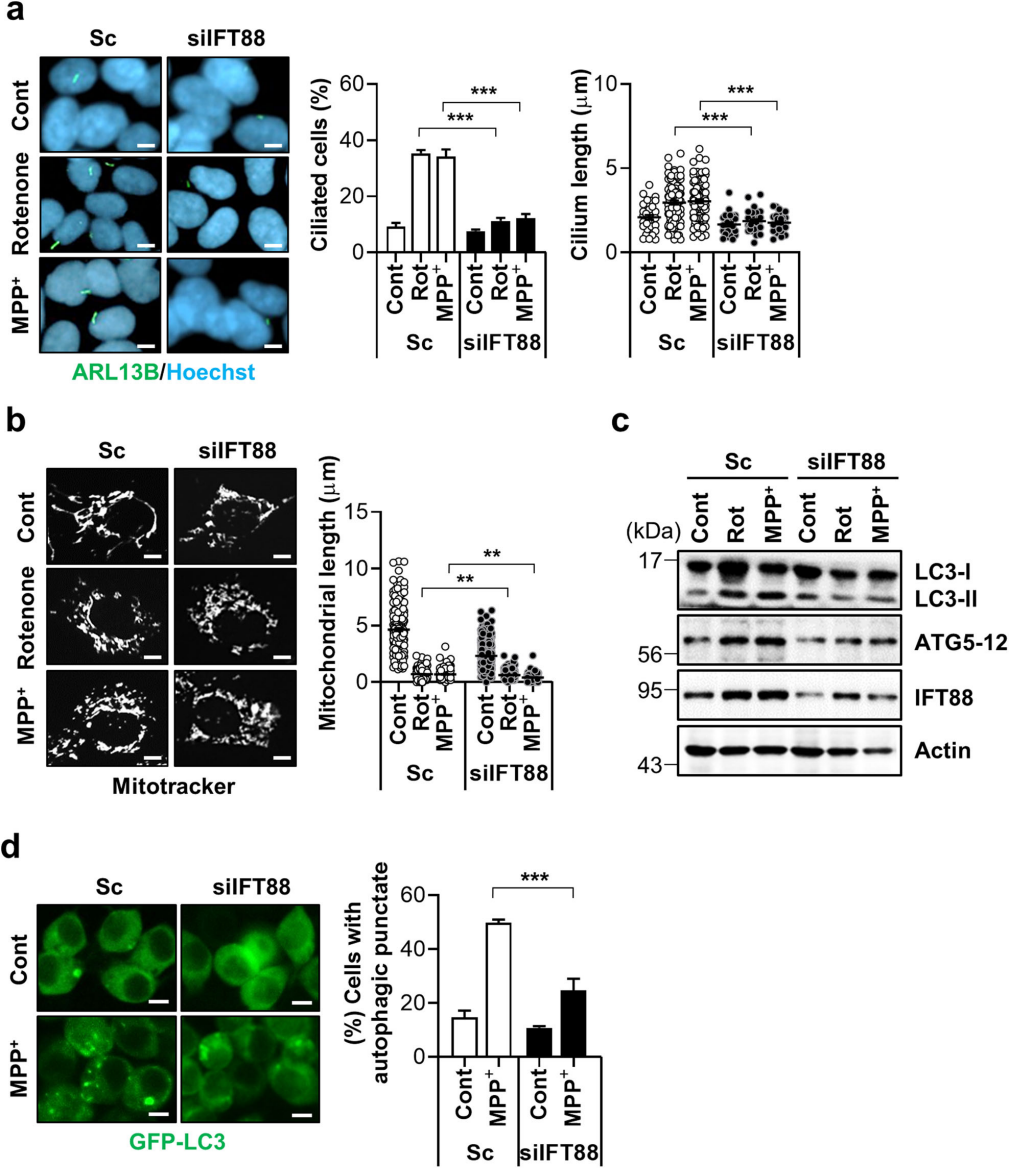
Ciliogenesis mediates mitochondrial stress-induced autophagy. **a**, **b** SH-SY5Y cells transfected with si*IFT88* were further treated with rotenone (200 nM) or MPP^+^ (5 mM) for 24 h. **a** Primary cilia were immunostained with ARL13B (green) and the nucleus was stained with Hoechst 33342 dye (blue). Cilia were measured in about 200 cells per group. **b** Mitochondria were stained with MitoTracker (white). Scale bar, 5 μm. **c** Inhibition of ciliogenesis with *IFT88* siRNA (si*IFT88*) blocked rotenone- and MPP^+^-induced autophagy in SH-SY5Y cells. **d** SH-SY5Y/GFP-LC3 cells transfected with scrambled control siRNA (Sc) or siRNA against *IFT88* (si*IFT88*) were further treated with rotenone (200 nM) and MPP^+^ (5 mM) for 24 h and then cells with autophagic punctate were counted under fluorescence microscopy. Scale bar, 10 μm. Data are the mean ± SEM. **p < 0.01, ***p < 0.005 between indicated groups determined by ANOVA followed by a post hoc LSD test.

**Fig. 6:**
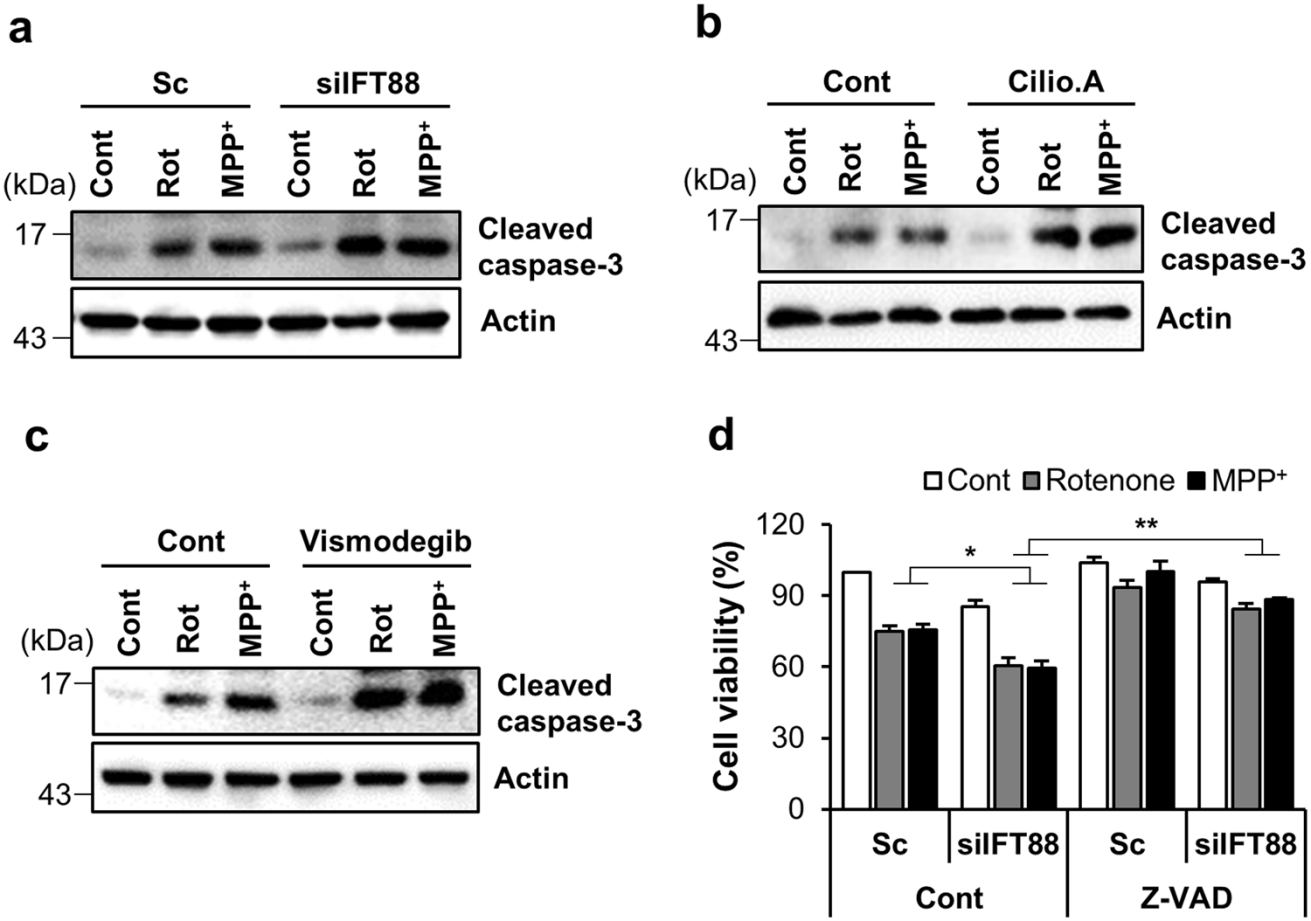
Mitochondrial stress-induced ciliogenesis promotes cell survival. **a**–**c** Accelerated rotenone- and MPP^+^- induced cell death in SH-SY5Y cells with defective ciliogenesis. **a** SH-SY5Y cells transfected with Sc or si*IFT88* were treated with rotenone (200 nM) and MPP^+^ (5 mM) for 24 h. Then the cells were further analyzed by Western blotting with cleaved caspase-3 antibody. **b**, **c** A blockade of ciliogenesis was induced by with (**b**) ciliobrevin A1 (Cilio.A, 10 μM) or (**c**) vismodegib (5 μM) treatment in SH-SY5Y cells and caspase-3 activation was examined by Western blotting with cleaved caspase-3 antibody. **d** SH-SY5Y cells were transfected with siRNA against *IFT88* and further treated with rotenone (200 nM) or MPP^+^ (5 mM) for 24 h in the presence or absence of the pan caspase inhibitor Z-VAD (40 μM). Cell viability was measured by CCK-8 flow cytometric analysis. Data are the mean ± SEM (*n* = 3 per group). *p < 0.05, **p < 0.01 between the indicated groups determined by ANOVA followed by a post hoc LSD test.

Reciprocal regulation between primary cilia and autophagy has been reported previously^6^. Primary cilia stimulate autophagy through the inhibition of mTOR signaling in kidney tubular epithelial cells^11^. We thus tested the possibility that enhanced ciliogenesis conversely regulates autophagy during mitochondrial stress. IFT88/polaris is a major component of the IFT-B protein complex that mediates the antegrade IFT, and a lack or hypomorphic mutation of *IFT88* disrupts cilia assembly^23^. Consistently, the downregulation of IFT88 sufficiently blocked rotenone- or MPP^+^-induced ciliogenesis (Fig. 5a). Notably, in IFT88 depleted cells, mitochondrial length was slightly decreased by treatment with either rotenone- or MPP^+^ (Fig. 5b). Furthermore, a blockade of inducible ciliogenesis by the depletion of IFT88 also suppressed the rotenone/MPP^+^-elicited autophagic activation by reducing LC3-α accumulation (Fig. 5c, d). Since both the primary cilia and autophagy are involved in promoting cell survival under stressful conditions^24,25^, we tested the effects of a ciliogenesis blockade on mitochondrial stress-induced cell death. Consistent with this notion, cells with defective ciliogenesis by resulting from the depletion of IFT88 expression were more prone to mitochondrial inhibitor-induced apoptosis, as determined by caspase-3 cleavage (Fig. 6a). Additionally, treatment with an antagonist of the Hh signaling pathway, vismodegib or a cytoplasmic dynein inhibitor, ciliobrevin A1 also enhanced caspase activation in rotenone and MPP^+^-treated cells (Fig. 6b, c). We also found that the application of a pan caspase inhibitor, zVAD suppressed mitochondrial-toxin-mediated cell death and mitochondrial fragmentation in IFT88 knockdown cells (Fig. 6d and Supplementary Fig. 12). Taken together, these data underscore the important role of ciliogenesis in the coordination of cellular responses to promote cell survival.

### Enhanced ciliogenesis in dopamine neurons promotes autophagy and neuronal survival in an MPTP-induced Parkinson’s disease model

**Fig. 7:**
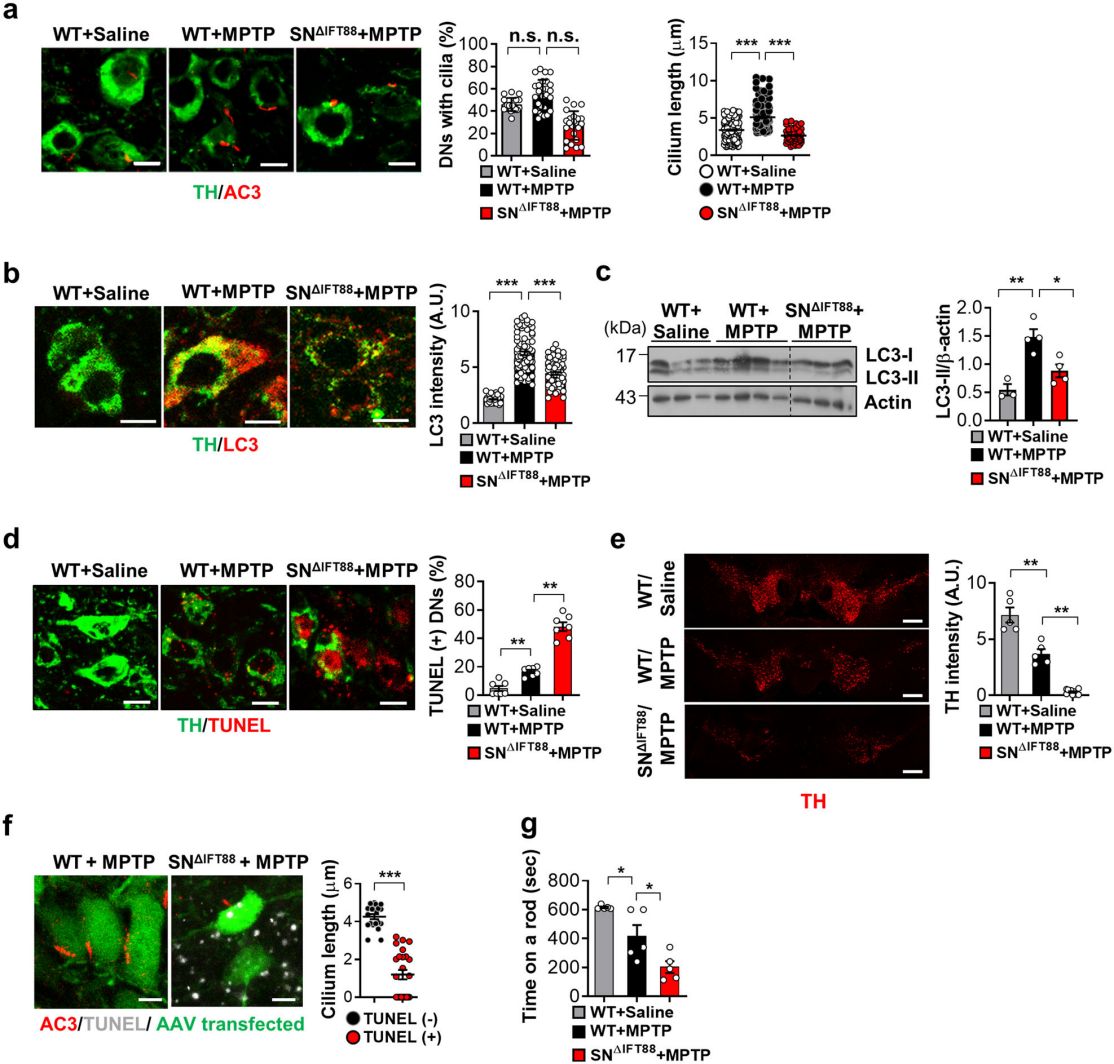
Enhanced ciliogenesis in the substantia nigra dopamine neurons in the mice model of MPTP-induced Parkinson’s disease. **a** Ciliary elongation in substantia nigra (SN) dopamine neurons (DNs) in the mouse after a single intraperitoneal administration of MPTP (30 mg/kg body weight) and blockade of cilia elongation with SN IFT88 knockdown. Two weeks before MPTP injection, the WT mice were injected with GFP-AAV and SN^∆IFT88^ mice were injected with *IFT88*-shRNA GFP-AAV into the bilateral SN. Primary cilia and DNs were stained using AC3 antibody and TH antibody. More than 100 TH-positive cells were analyzed in each animal. Scale bar, 10 μm. **b**, **c** Increased autophagy in SN DNs following MPTP treatment and blunted MPTP-induced autophagy in DNs with impaired ciliogenesis. Autophagy in DNs was evaluated by double staining with LC3 and TH antibodies and by LC3 immunoblotting. Scale bar, 10 μm. **d** Impaired ciliogenesis in the DNs enhances MPTP-induced apoptosis, as assessed by TUNEL staining. Scale bar, 10 μm. **e** MPTP treatment reduces the intensity of TH immunoreactivity in the SN pars compacta and this reduction is far greater in SN^ΔIFT88^ mice. Scale bar, 500 μm. **f** Cilia elongation occurs before DN death. TUNEL-negative neurons of WT mice have long cilia but TUNEL-positive neurons in SN^ΔIFT88^ mice have shorter or no cilia following MPTP treatment. Scale bar, 10 μm. **g** Motor function assessment using the rotarod test for 3 days after MPTP treatment in WT and SN^ΔIFT88^ mice (*n* = 3~6 per group). Data are presented as the mean ± SEM. *p < 0.05, **p < 0.01, ***p < 0.005 between the indicated groups determined by ANOVA followed by a post hoc LSD test (*n*.*s*. = not significant).

Mitochondrial abnormalities have been implicated in a wide range of human disorders, including neurodegenerative diseases such as Parkinson's disease (PD), and are considered to be a central event responsible for the progressive loss of dopamine (DA) neurons in the substantia nigra pars compacta (SN)^26^. Indeed, rotenone and 1-methyl-4-phenylpyridinium (MPTP), a prodrug of MPP^+^, are commonly used to induce experimental PD models^27^. Using MPTP-induced toxic PD models, we investigated the role of primary cilia in mitochondrial stress-related neuronal injury. Two weeks prior to MPTP injection, a group of the mice (6/16) received a microinjection of adeno-associated viruses (AAVs), that expressed *IFT88*-specific small hairpin RNA (shRNA) and GFP in a neuron-specific manner, into the bilateral SN to inhibit SN neuronal ciliogenesis (SN^ΔIFT88^ mice). Using this technique, we achieved successful AAV infection in SN DA cells, confirmed by examining GFP expression (Supplementary Fig. 13). The rest of the animals (10/16) received an intra-SN injection of a GFP-expressing AAV as a control. The midbrain was collected 3 days after the MPTP injections. Double staining of tyrosine hydroxylase (TH) in the mouse mid-brain to label the DA neuron, and type 3 adenylyl cyclase (AC3), to mark the neuronal primary cilia revealed a notable degree of ciliary elongation in the SN DA neurons at 3 days after MPTP injection. In contrast, MPTP-induced ciliary elongation was successfully blunted in DA neurons expressing *IFT88* shRNA (Fig. 7a). TH and LC3 co-staining showed that systemic MPTP treatment increases autophagy in dopaminergic neurons (Fig. 7b). MPTP-induced autophagic activation was significantly reduced in *IFT88* shRNA-expressing neurons (Fig. 7b). Consistently, LC3 immunoblotting showed that LC3-II expression in the SN area was increased after MPTP administration and the increased LC3-II expression by MPTP was significantly blunted in the *IFT88* shRNA-injected SN (Fig. 7c).

The assessment of DA-neuronal apoptosis using dual staining of TH and TUNEL (which shows apoptotic DNA fragmentation) revealed a dramatic increase in DA-neuronal apoptosis in MPTP-treated SN^ΔIFT88^ mice compared with the MPTP only-treated animals (Fig. 7d). MPTP treatment led to a significant reduction in the intensity of SN TH expression and this reduction was exaggerated in SN^ΔIFT88^ mice (Fig. 7e). We also conducted experiments in which *IFT88* shRNA-GFP-AAV was injected into the left-side SN and GFP-AAV was injected into the right-side SN. We compared TH intensity and neuronal death between the right and left SN. TH expression was profoundly suppressed and TUNEL-positive neurons were more frequently found in *IFT88* shRNA-injected side compared to the contralateral side (Supplementary Fig. 14). To address whether cilia elongation may occur before or during cell death, we conducted AC3 and TUNEL double staining. The SN neurons with long cilia in WT mice were not TUNEL-positive, whereas TUNEL-positive neurons in SN^ΔIFT88^ mice had shorter or no cilia (Fig. 7f). Therefore, cilia elongation occurs before DA neuronal death by mitochondria stress. Consistently, the numbers of TUNEL-positive SN neurons with shorter or no cilia increased at 7 days after MPTP administration when compared to those observed at 3 days post-treatment (Supplementary Fig. 15).

Finally, we assessed coordinated motor function using the rotarod performance test for 3 days after MPTP injections. The average time spent by the MPTP-treated mice on a rod was significantly shorter than that by the saline-injected mice (Fig. 7g). SN^ΔIFT88^ mice treated with an MPTP injection exhibited severe motor dysfunction. These in vivo findings reveal a protective role of the primary cilium against MPTP-induced dopamine neuronal loss and motor disability.

## Discussion

The present study demonstrates the interplay between two seemingly unrelated organelles: the primary cilium and mitochondria. Disruption of mitochondrial respiratory function stimulates ciliary growth in different types of cells. Moreover, mitochondrial fission stimulates while mitochondrial fusion suppresses cilia formation, thus demonstrating a novel regulatory function for mitochondria dynamics in primary ciliogenesis. Based on the result of this study, we propose that both mtROS- and AMPK-driven autophagy are major mechanisms underlying mitochondrial stress-induced ciliogenesis (Figs. 3 and 4). Excessive fragmented mitochondria generate higher levels of ROS, which play a role in various biological pathways. AMPK is a redox-sensitive cellular energy sensor activated by oxidative stress and ATP depletion^16^. We found that mitochondrial fragmentation induced by either *OPA1* knockdown or administration of mitochondrial toxins activates AMPK phosphorylation, which subsequently triggers autophagy by inhibiting the mTOR pathway (Fig. 4). Blockade of these mitochondrial fission–mtROS-AMPK-autophagy activation pathways prevents mitochondria stress-related cilia elongation. Interestingly, both autophagy activation and the assembly of primary cilium are induced by serum deprivation, suggesting a bidirectional interplay between autophagy and primary cilia. Tang et al. demonstrated that autophagy triggers the biogenesis of primary cilium^7^. On the other hand, Pampliega et al. showed that genetic inhibition of autophagy enhances cilia-associated signaling such as Hh signaling under the serum starvation condition in *Atg5-*KO MEF cells^6^. Some of these opposite effects can be attributed to difference in autophagic degradation targets. Under the nutrient rich condition, basal autophagy suppresses primary cilia by removing the essential ciliary protein IFT20^6^. However, under the serum deprivation condition, IFT proteins are required to form primary cilia, and the autophagy target changes to promote the degradation of OFD1 and subsequently increase the growth of primary cilia^7,28,29^. In this study, we found that autophagy induced by mitochondrial stress also increased the formation of primary cilia by promoting the degradation of ODF1 under nutrient rich conditions. In line with our findings, serum starvation and specific compounds (PPAR-α agonist, sertraline, BIX-01294, etc.) stimulate ciliary growth by the degradation of OFD1 protein through autophagic mechanisms^7,28,30^. Somatic mTOR gain-of function mutations impair ciliogenesis in the developing brain through compromised autophagic removal of OFD1, leading to focal malformation in cortical development^21^.

In the current study, we found that inhibition of ciliogenesis sensitizes neuronal cells to mitochondrial toxin-induced cell death (Figs. 6 and 7). These protective actions of primary cilia are mediated, at least in part, through accelerated autophagy, as a cell’s ability to grow cilia is closely related to its capacity for inducible autophagy. Moreover, ciliogenesis can be regulated by inner cellular stresses such as oxidative stress and the presence of the alkylating agent, cisplatin^31,32^. In support of this notion, it has been recently reported that dysfunction of primary cilia by loss of ciliary proteins such as PCM1 and Tctn3 increases apoptotic cell death in glioblastoma or caused neuronal apoptosis in mice^33,34^. In contrast, the activation of Hh signaling, which depends on primary cilia, decreases ischemic injury and improves neurological function after stroke^35^. Cilia-mediated Hh signaling is able to activate autophagy^6^. Primary cilia reciprocally regulate autophagy, which has protective effects by eliminating damaged mitochondria under oxidative stress condition^11,36,37^. We also found that the blockage of primary cilia sensitized the cells to mitochondrial stress-induced neuronal cell death. Together with our findings, these results suggested that ciliogenesis may be an important adaptive mechanism for mitochondrial stress insults in mammalian cells.

Our experimental evidence indicates potential roles for the primary cilia in PD pathology. Dramatic elongation of the cilia is evident in the SN neurons of the PD animal model. Moreover, SN neurons with compromised ciliogenesis fail to induce autophagy and are prone to apoptosis upon exposure to MPTP. Impaired ciliogenesis in SN DA neurons leads to severe motor dysfunction at the acute treatment phase although its long-term, delayed effects on the progression of PD requires further study. Interestingly, ciliary elongation was also observed in striatal neurons in toxic PD models^38^, which receive DA neuronal input from the SN, suggesting that primary cilia has additional roles in the pathology of PD. Common PD-associated genetic mutations in leucine-rich repeat kinase 2 (LRRK2) are associated with abnormal microtubule organization^39^. According to this notion, it was recently reported that overexpression of a PD-associated pathogenic LRRK2 mutant (R1441C) results in defective cilia in mouse striatum and reduces Hh signaling^40^.

Taken together, our present findings extend the knowledge on repertoire of cilia-related biological functions and related diseases and also offer a potential new therapeutic avenue for PD.

## Materials and methods

### Reagents

Bafilomycin A1 (B1793), Carbonyl cyanide m-chlorophenyl hydrazine (CCCP, C2759), rotenone (R8875), 1-methyl-4-phenylpyridinium (MPP^+^, D048), doxycycline (D9891), and N-acetyl-cysteine (NAC, A9165) were purchased from Sigma-Aldrich (St. Louis, MO). 3-(2,4-dichloro-5-methoxyphenyl)-2,3-dihydro-2-thioxo-4(1H)-quinazolinone (Mdivi-1, BML-CM127) was purchased from Enzo Life Sciences (Farmingdale, NY). Ciliobrevin A1 (#4529) and Torin-1 (#4247) were purchased from Tocris Bioscience (Bristol, UK). Vismodegib (GDC-0449) was purchased from Selleckchem (Munich, Germany). zVAD-FMK (FMK001) was purchased from R&D systems (Minneapolis, MN). 1-methyl-4-phenyl-1,2,3,6-tetrahydropyridine (MPTP, HY-15608) was obtained from MedChem Express (Monmouth Junction, NJ).

### Cell lines

SH-SY5Y neuroblastoma cells were obtained from ATCC (Manassas, VA). Human telomerase-immortalized retinal pigmented epithelial (RPE) cells were kindly provided by Dr. Jun Kim (KAIST, South Korea). Wild-type (WT) MEFs, as well as *Drp1* and *OPA1* knockout MEFs were generously provided by Dr. Katsuyoshi Mihara (Kyushu University, Japan) and Dr. Joo-Yong Lee (Chungnam National University, Korea). MEFs including a doxycycline-induced deletion of *ATG5* (M5-7 cells) were kindly provided by Dr. Noboru Mizushima (Tokyo University, Japan), with ATG5 depletion being induced by maintaining the cells in doxycycline (1 μg/ml) containing medium, for 4 days. *AMPK* double knockout (*AMPK* DKO) MEFs were kindly provided by Dr. Benoit Viollet (Université Paris Descartes, France) and maintained in doxycycline-containing culture medium. To generate stable cell lines, SH-SY5Y cells were transfected with pEGFP-LC3 (SY5Y/GFP-LC3 cells), pEGFP-TFEB (SY5Y/GFP-TFEB cells), and pMito-HyPer (SY5Y/Mito-HyPer) using Lipofectamine 2000 in accordance with the manufacturer’s protocol (#11668019, Thermo Fisher Scientific, Waltham, MA). Transfectants were selected by growth in medium containing 1 mg/ml of G418 (#10131027, Thermo Fisher Scientific) for 7 days. After single cell dropping, the stable clones were selected under a fluorescence microscope (IX71, Olympus, Tokyo, Japan).

### Animals

C57BL/6 male mice (8 weeks of age) were purchased from Orient Bio (Seongnam, Korea) and housed under a controlled temperature (22 ± 1 °C) and a 12 h light-dark cycle (lights on 8 AM) with free access to food and water. All animal procedures were approved by the Institutional Animal Care and Use Committee of the Asan Institute for Life Science (Seoul, Korea).

### Induction of mitochondrial stress

We induced mitochondrial stress by treating cells with mitochondrial oxidative phosphorylation inhibitors such as rotenone, CCCP and MPP^+^ at the indicated doses or by inhibiting mitochondrial fusion through the depletion of the mitochondrial fusion factor OPA1. In mice, mitochondrial stress in substantia nigra (SN) dopamine (DA) neurons was induced by a single intraperitoneal injection of MPTP (30 mg per kg body weight).

### Gene knockdown studies

For gene expression knockdowns, cells were transfected with previously validated siRNAs targeting human *OPA1* (5′-cuggaaagacuaguguguu-3′), *Drp1* (5′-gagguuauugaacgacuca-3′), *ATG5* (5′-gcaacucuggaugggauug-3′), *IFT88* (5′-ccgaagcacuu-aacacuua-3′), *AMPK-α1/α2* (5′-augaugucagauggugaauuu-3′), and mouse *OPA1* (5′-gaaacuuucuccaauuaaauu-3′) using Lipofectamine 2000. The siRNAs were synthesized from Genolution (Seoul, Korea). At 48 h post-transfection, the cells were further treated with the indicated reagents. To induce IFT88 knockdown in the SN neurons of mice, a 500 nl mixture (1:1 ν/ν) of adeno-associated virus (AAV) (1 × 10^12^ genome copies/ml) expressing Cre-recombinase and EGFP under control of the synapsin promotor (AAV-DJ-hSyn-Cre:EGFP) and AAV expressing small hairpin RNA (shRNA: sense sequence: ttggagcttattacattgata) specific to mouse *IFT88* in a Cre-dependent manner (AAV-DJ-DIO-TATAlox-EYFP-sh*IFT88*) was bilaterally microinjected to the SN areas using stereotaxic surgery (coordination: 2.8 mm back from the bregma, 0.05 mm lateral to the sagittal sinus and 4.3 mm deep from the skull surface). Control animals were injected with 500 nl of AAV-DJ-hSyn-Cre:EGFP. AAVs were infused at the rate of 50 nl/min over 10 min using a Hamilton microsyringe (#7634-01, Hamilton Robotics, Reno, NV). A successful virus injection and IFT88 knockdown was determined by examining EGFP expression and cilia loss in the SN area, respectively. In a separate study, *IFT88* shRNA-AAV was injected into the left-side SN and GFP-AAVs was injected into the right-side SN of mice 2 weeks before MPTP administration.

### Cilia staining and counting

For the staining of primary cilia, cells were washed with cold phosphate buffered saline (PBS) and fixed with 4% (w/v) paraformaldehyde (PFA), dissolved in PBS containing 0.1% (v/v) Triton X-100. Subsequently, the cells were blocked with PBS containing 1% bovine serum albumin (BSA), and incubated overnight at 4 °C with primary antibodies against acetylated α-tubulin (1:1000, T7451, Sigma-Aldrich) or ARL13B (1:1000, 17711-1-AP, Proteintech) in 1% BSA. After washing, the cells were incubated with Alexa Fluor 488 or 555-conjugated secondary antibodies at room temperature (RT) for 1 h. Before mounting, the cells were treated with Hoechst 33342 dye (1:10,000, H3570, Thermo-Fisher) for nuclear staining. Cilia images were observed using a fluorescence microscope. Cilia were counted in about 200 cells under each experimental condition (*n* = 3). The ciliated cell percentage was calculated as (total number of cilia/total number of nucleus at each image) × 100. Cilia lengths were measured using the Free-hand Line Selection Tool of Cell Sense Standards software (Olympus Europa Holding GmbH, Hamburg, Germany) and the average cilium lengths were calculated. Analysis of graph data was performed with GraphPad Prism 8 (GraphPad Software, San Diego, CA).

For the cilia staining in the DA neurons of SN in mice, the animals were perfused with 50 ml of 4% PFA via the left ventricle under anesthesia with 40 mg/kg Zoletil and 5 mg/kg Rompun. Whole brains were collected, post-fixed with 4% PFA overnight, and dehydrated in 30% sucrose solution until the tissues sank to the bottom of the container. Coronal brains including the SN with reference to Allen Mouse Brain Atlas were sectioned 30 μm thick using a cryostat (Leica, Wetzlar, Germany) and stored at −70 °C. Brain slices were blocked with 3% BSA at RT for 1 h, incubated with anti-type 3 adenylyl cyclase (AC3) antibody (1:500, rabbit, sc588, Santa Cruz Biotechnology, Dallas, TX) at 4 °C for 48 h and then treated with secondary anti-rabbit antibody (1:1000, Thermo-Fisher) at RT for 1 h. For DA neuron staining, brain sections were further subjected to tyrosine hydroxylase (TH) staining. Brain sections were blocked with 2% normal horse serum in PBS at RT for 1 h, incubated with anti-TH antibody (1:400, chicken, ab76442, Abcam, Cambridge, UK) at 4 °C overnight and anti-chicken secondary antibody (1:1000, Thermo-Fisher) at RT for 1 h. Before mounting, slices were incubated with DAPI (1:10,000, 5 min) for nuclear staining. Immunofluorescence was detected and imaged using a confocal microscopy (Carl Zeiss 780, Germany). Cilia lengths in TH^+^ DA neurons in the SN were measured using Image J. Five brain sections including the SN were analyzed each animals.

### Evaluation of mitochondrial fission and fusion

Mitochondrial dynamics in cells were examined via morphology. For the staining of mitochondria, cells were fixed with 4% PFA and then treated with MitoTracker probe (100 nM, M7512, Thermo-Fisher) for 30 min. Mitochondrial images were obtained using a fluorescence microscope (IX71, Olympus, Japan). Mitochondrial lengths were measured using the Free-hand Line Selection Tool of Cell Sense Standards software (Olympus Europa Holding GmbH, Hamburg, Germany). The mean length of the mitochondria was determined by selection of 20–30 linearized and unconnected filament-like mitochondria per cell using a tool provided by the Cell Sense Standards Software (*n* = 3 independent experiments). And the images of at least 10 randomly selected cells per individual were analyzed and digitized using GraphPad Prism 8 (GraphPad Software, San Diego, CA).

### Western blot analysis

Cell lysates were prepared in 2x Laemmli sample buffer (62.5 mM Tris-HCl, pH 6.8, 25% [v/v] glycerol, 2% [w/v] SDS, 5% [v/v] β-mercaptoethanol, and 0.01% [w/v] bromophenol blue) (#161-0737, Bio-Rad, Hercules, CA). After separation in 10–12% SDS-PAGE, the proteins were transferred onto PVDF membrane (#162-0177, Bio-Rad). The membranes were then incubated with the following primary antibodies: OPA1 (#612606, BD, San Jose, CA), Drp1 (#611738, BD), ATG5 (ab54033, Abcam, Cambridge, UK), IFT88 (13967-1-AP, Proteintech, Chicago, IL), OFD1 (22851-1-AP, Proteintech), LC3 (NB100-2220, Novus Biologicals, Littleton, CO or L7543, Sigma-Aldrich), p62 (#5114, Cell Signaling Technology, Danvers, MA), AMPK (#1596, Epitomics, Burlingame, CA), phospho-AMPK (T172) (#2535, Cell Signaling Technology), cleaved caspase-3 (#9661S, Cell Signaling Technology) and actin (MAB1501, Millipore, Temecula, CA). For protein detection, the membranes were incubated with horseradish peroxidase (HRP)-conjugated secondary antibodies (Pierce, Rockford, IL). Chemiluminescent signals were developed using Clarity Western ECL substrate (W3680-010, Bio-Rad). Densitometry was performed on scanned immunoblots using the AE-9300 Ez-Capture MG Hours Image Saver HR image capture tool (WSE-7120L, ATTO, Tokyo, Japan). Each protein expression level was normalized to that of actin.

### Determination of mitochondrial ROS

Mitochondria-specific ROS levels were assessed using a HyPer protein system. The pHyPer-dMito vector encoding mitochondria-targeted HyPer (Mito-HyPer) was obtained from Eyrogen (San Diego, CA). SH-SY5Y cells stably expressing Mito-HyPer were transfected with scrambled or *OPA1* siRNA for 72 h in the presence or absence of NAC (1 mM). In a separate study, cells were treated with rotenone (200 nM) for 24 h with or without NAC. Cellular fluorescence intensities were monitored by a fluorescence plate reader (excitation 500 nm/emission 516 nm) (Victor X3, Perkin-Elmer Life Sciences, Waltham, MA) or under fluorescence microscopy. The relative ROS ratio was presented as the fluorescence intensity of *OPA1* siRNA- or rotenone-treated samples divided by that of the control samples.

### Measurement of mitochondrial membrane potential

Mitochondrial membrane potential was measured with a unique fluorescent cationic dye, JC-1 (5,5′,6,6′-tetrachloro-1,1′,3,3′-tetraethylbenzimidazolylcarbocyanine iodide, BD Biosciences) that detects signal loss for mitochondrial membrane potential. The fluorescence intensity was measured using the Attune NxT flow cytometer (Thermo-Fisher) at excitation and emission wavelengths of 488 and 530 nm, respectively, for the monomeric form as well as at 561 and 585 nm for the J-aggregate forms.

### Autophagy analysis

SY5Y/GFP-TFEB cells were seeded on 24-well plates and treated with Torin-1 (1 μM for 1 h), rotenone (200 nM for 24 h), or MPP^+^ (5 mM for 24 h). Cells with nuclear TFEB were captured and counted under a fluorescence microscope. SY5Y/GFP-LC3 cells were plated on 24-well plates and transfected with *IFT88* siRNA for 48 h and additionally treated with MPP^+^ (5 mM) for 24 h. Cells were washed with PBS, and fixed with 4% PFA. Autophagy puncta with GFP-LC3 were also captured and counted under a fluorescence microscope. In mice, autophagy in SN dopamine neurons was evaluated with LC3 and TH double immunofluorescent staining. Brain slices were subjected to serial immunofluorescent staining of LC3 and TH staining. For LC3 staining, brain slices were incubated with 3% normal donkey serum at RT for 1 h and then with rabbit anti-LC3 antibody (1:200, #ab51520, Abcam, Cambridge, UK) at 4 °C overnight. TH staining was performed as described for the cilia staining. Five brain sections including the SN were examined in each animal. For LC3 western blotting in mice, the SN was collected from 1 mm-thick midbrain slice using a punch biopsy technique. Tissues were immediately frozen in liquid nitrogen and stored at –70 °C until protein extraction.

### Measurement of cell survival and death

SH-SY5Y cells were transfected with *IFT88* siRNA using Lipofectamine and additionally treated with rotenone (200 nM) or MPP^+^ (5 mM) for 24 h. In a separate experiment, the cells were treated with rotenone or MPP^+^ in the presence or absence of ciliobrevin A1 (10 μM) for 24 h. Cell viability was assayed using a Cell Counting Kit-8 kit (CK04-11, Dojindo Laboratories, Kumamoto, Japan) following the manufacturer’s protocol. Apoptotic cell death was assessed using western blotting of cleaved caspase-3. In animals, survival of DA neurons was assessed by counting the numbers of neurons with TH expression. On the other hand, apoptotic death of DA neurons was determined by TH and TUNEL double staining. TUNEL staining was performed using a fluorometric TUNEL detection kit (#11684795910, Roche Applied Science, Indianapolis, IN or C10619, Thermo-Fisher, Waltham, MA). Briefly, brain slices were prepared and stained using TH or AC3 antibody as described for cilia staining and prior to TUNEL staining. The brain sections were permeabilized with 0.2% Triton X-100 in 0.1% sodium citrate at 4 °C for 2 min, and then incubated with the provided fluorescein-conjugated TUNEL reaction mixture in a humidified chamber at 37 °C for 1 h in the dark. Fluorescent images were obtained using a confocal microscope and the numbers of TH^+^ dopamine neurons and dopamine neurons with TUNEL signal positivity were counted in 5 brain sections of each animal. The cilia lengths in neurons with or without TUNEL signals and the cell percentages with TUNEL intensity among transfected SN neurons were analyzed in the SN of mice injected with GFP-AAV or sh*IFT88*-GFP-AAV at 3 and 7 days after MPTP administration.

### Motor function test

Sensorimotor coordination ability was determined with the rotarod performance test. Briefly, 8-week-old male mice were trained for 2 weeks prior to MPTP injection to balance on a rotating rod (B.S Technolab, Seoul, Korea). The rod accelerated from 4 to 40 rpm within 1 min and then constantly rotated at 40 rpm. Training was performed for 10 min per trial, two trials per session, and three sessions per week. Following MPTP injection, mice were placed daily on a rod for 3 days and the latency to falling was recorded. The average of six trials (two trials per day for 3 days) is presented in the results.

### Statistical analysis

Statistical analyses of the results were performed by one-way analysis of variance (ANOVA) followed by a post hoc LSD test or an unpaired Student’s *t*-test using Origin software (San Clemente, CA) or SPSS version 23 (IBM Analytics, North Castle, NY). Data were obtained from at least three independent experiments, and presented as the mean ± the standard error of the mean (SEM). Significance was defined as *p* < 0.05.

## Supplementary information

Suppl. Fig. Legend

Suppl. Fig. 1

Suppl. Fig. 2

Suppl. Fig. 3

Suppl. Fig. 4

Suppl. Fig. 5

Suppl. Fig. 6

Suppl. Fig. 7

Suppl. Fig. 8

Suppl. Fig. 9

Suppl. Fig. 10

Suppl. Fig. 11

Suppl. Fig. 12

Suppl. Fig. 13

Suppl. Fig. 14

Suppl. Fig. 15

## Data Availability

All of the data generated and analyzed in this study are included in this published article.
