# Supplementary material for: Primary cilia mediate mitochondrial stress responses to promote dopamine neuron survival in a Parkinson’s disease model
Source: Cell Death Dis. 2019 Dec 16;10(12):952. doi: 10.1038/s41419-019-2184-y (PMC6915731; doi:10.1038/s41419-019-2184-y)
Supplement: Supplementary file 1 — Suppl. Fig. Legend [file 41419_2019_2184_MOESM1_ESM.docx]

**Supplementary Figure Legends**

**Figure S1. Examination of *Drp1* and *OPA1* gene knockdown**

Successful knockdown of *Drp1* and *OPA1* using siRNA in SH-SY5Y cells was confirmed by immunoblotting. SH-SY5Y cells transfected with scrambled siRNA (Sc), siRNA for *Drp1* (si*Drp1*) or siRNA for *OPA1* (si*OPA1*) for 3 days were analyzed by Western blotting with the indicated antibodies.

**Figure S2. Effects of mitochondrial fusion and fission on ciliary growth in RPE cells**

Mitochondrial fission induced by *OPA1* siRNA (si*OPA1*) treatment enhanced ciliogenesis whereas mitochondrial fusion caused by *Drp1* siRNA (si*Drp1*) had no significant effects on ciliary growth. SH-SY5Y cells transfected with si*Drp1* or si*OPA1* were stained with a MitoTracker (white). Cilia were immunostained with acetylated α-tubulin (AT) (red) and nuclei were counterstained with Hoechst 33342 dye (blue). Representative mitochondria and cilia images are presented. Cilia were measured in about 200 cells per group. Data are the mean ± SEM. ***p* < 0.01, ****p* < 0.005 vs. a scrambled siRNA (Sc)-treated group determined with ANOVA followed by a *post-hoc* LSD test. Scale bar, 5 μm.

**Figure S3. Effects of serum starvation on ciliary growth in *OPA1*- and *Drp1* KO MEF cells**

Wild type (WT) mouse embryonic fibroblasts (MEFs) cells, *OPA1*-knockout (KO) and *Drp1*-KO MEFs cells grown under serum-free (SF) condition were immunostained with ARL13B (green), and Hoechst 33342 dye (blue). Representative cilia images are presented. Cilia measurement data were obtained from about 200 cells per group and the experiments were repeated at least three times. Data are the mean ± SEM. **p* < 0.05, ****p* < 0.005 vs. WT MEF cells determined by ANOVA followed by a *post-hoc* LSD test. Scale bar, 5 μm.

**Figure S4. Effect of NAC treatment on *OPA1* knockdown- or rotenone-induced ciliogenesis in RPE cells**

RPE cells were transfected with scrambled control siRNA (Sc) or *OPA1* siRNA (si*OPA1*). After 2 days, the cells were treated with NAC (1 mM) for 24 hr. RPE cells were treated with rotenone (200 nM) in the presence or absence of NAC (1 mM) for 24 hr. Primary cilia were immunostained with acetylated α-tubulin (AT) (red) and the nucleus was counterstained with Hoechst 33342 dye (blue). Ciliary measurements were performed for about 200 cells per group. Data are the mean ± SEM. ***p* < 0.01, ****p* < 0.005 between the indicated groups determined by ANOVA followed by a *post-hoc* LSD test. Scale bar, 5 μm.

**Figure S5. Effect of NAC treatment on mitochondrial stress-induced fission in SH-SY5Y cells**

SH-SY5Y cells were transfected with siRNA against scrambled control (Sc) or *OPA1* (si*OPA1*). After 2 days, the cells were treated with NAC (1 mM) for 24 hr. SH-SY5Y cells were treated with rotenone (200 nM) in the presence or absence of NAC (1 mM) for 24 hr. Mitochondrial morphology was assessed by MitoTracker staining (white). Data are the mean ± SEM. ****p* < 0.005 between the indicated groups determined by ANOVA followed by a *post-hoc* LSD test. Scale bar, 5 μm.

**Figure S6. Examination of *AMPK* knockdown**

Successful AMPK knockdown using siRNA in SH-SY5Y cells which prevented rotenone and MPP^+^-induced AMPK activation. SH-SY5Y cells were transfected with Sc or *AMPK* siRNA (si*AMPK*) and further treated with rotenone (200 nM) or MPP^+^ (5 mM) for 24 hr. AMPK activity was assessed based on the phosphorylation of this kinase at T172. Representative immunoblots are shown.

**Figure S7. Mitochondrial fission-induced ciliogenesis is blunted in *AMPK* double knockout (DKO) MEFs**

**a.** The *AMPK* knockout was confirmed by Western blotting with phosphorylated AMPK (p-AMPK) (T172) antibody. **b.** Wild type (WT) and *AMPK* *α1/α2* double-knockout (*AMPK* DKO) MEF cells were transfected with Sc or *OPA1* siRNA. After 3 days, the cells were immunostained with ARL13B (green) or Hoechst 33342 dye (blue). **c.** WT and *AMPK* DKO MEF cells were treated with rotenone (200 nM) or MPP^+^ (5 mM) under serum-free (SF) condition for 24 hr. Then primary cilia were immunostained with ARL13B and the nucleus were counterstained with Hoechst 33342 dye. Primary cilia were measured in about 200 cells per group. **d.** WT and *AMPK* DKO MEF cells treated rotenone (200 nM) or MPP^+^ (5 mM) were stained with MitoTracker (white). Data are the mean ± SEM. **p* < 0.05, ****p* < 0.005 between the indicated groups determined by ANOVA followed by a *post-hoc* LSD test. Scale bar, 5 μm.

**Figure S8. Inhibition of mitochondrial stress-induced autophagic flux in autophagy inhibitor treated SH-SY5Y cells**

SH-SY5Y cells were treated with rotenone (200 nM) or MPP^+^ (5 mM) with or without Bafilomycin A1 (10 nM) for 24 hr. Autophagic flux was assessed by LC3-II using Western blotting.

**Figure S9. Effect of autophagic inhibition on mitochondrial morphology in mitochondrial stress-induced *ATG5*-deficient MEF cells**

MEFs under doxycycline (Dox)-induced ATG5 depletion (M5-7 cells) were treated to rotenone (200 nM) and MPP^+^ (5 mM) for 24 hr in the presence or absence of Dox. Then the cells were stained with MitoTracker (white). Representative mitochondrial images are presented. Scale bar, 5 μm. Data were obtained from about 200 cells per group and experiments were repeated at least three times. Data are the mean ± SEM. ****p* < 0.005 vs. untreated controls determined by ANOVA followed by a *post-hoc* LSD test.

**Figure S10. Mitochondrial stress-induced OFD1 depletion is suppressed by autophagy inhibitor**

SH-SY5Y cells were treated with MPP^+^ (5 mM) with or without Bafilomycin A1 (10 nM) for 24 hr, then the cells were analyzed by Western blotting with the indicated antibodies.

**Figure S11. Autophagy activation protects from mitochondrial fission in SH-SY5Y cells**

SH-SY5Y cells were treated with Torin-1 (10 nM) for 1 hr or with rotenone (200 nM) or MPP^+^ (5 mM) for 24 hr. Mitochondria were stained with a MitoTracker (white). Representative images are presented. Data are the mean ± SEM. ****p* < 0.005 vs. untreated controls determined by ANOVA followed by a *post-hoc* LSD test. Scale bar, 5 μm.

**Figure S12. Inhibition of cell death prevents mitochondrial stress-induced fission in SH-SY5Y cells**

**a.** SH-SY5Y cells were transfected with scrambled (Sc) or *IFT88* siRNA and further treated with rotenone (200 nM) or MPP^+^ (5 mM) for 24 hr in the presence or absence of the pan caspase inhibitor Z-VAD (40 μM). Then mitochondria morphology was determined by MitoTracker staining under a fluorescence microscope. **b.** SH-SY5Y cells treated with rotenone (200 nM) or MPP^+^ (5 mM) were further exposed to ciliobrevin A1 (Cilio.A, 10 μM) in the presence or absence of Z-VAD (40 μM). Then mitochondria morphology was determined by MitoTracker staining under a fluorescence microscope. Data are the mean ± SEM. **p* < 0.05, ****p* < 0.05 vs. between the indicated groups determined by ANOVA followed by a *post-hoc* LSD test. Scale bar, 5 μm.

**Figure S13. Confirmation of successful AAV transfection in dopamine neurons of the substantia nigra**

Successful expression of GFP-*IFT88* shRNA-AAV or GFP-AAV in DA neurons of the SN was examined by double immunostaining of GFP (green) and tyrosine hydroxylase (TH, red). Scale bar, 500 μm.

**Figure S14.** **Comparisons of TH, TUNEL, and cilia immunostaining between the left-sided SN with IFT88 knockdown and the right-sided SN as a control**

**a**. TH/GFP double immunostaining. **b.** AC3/TUNEL/GFP triple immunostaining in the SN of mice injected with saline or MPTP (30 mg/kg) for 3 days before sacrificed. Two weeks before saline or MPTP injections, mice were injected with GFP-AAV in the right-sided SN and shIFT88-GFP-AAV in left-sided SN, respectively. Scale bars, 500 μm and 25 μm (magnified images).

**Figure S15.** **MPTP-induced dopamine neuron death in the SN of mice at 3 and 7 days after MPTP injections**

AC3, TUNEL and GFP triple immunostaining in the SN of mice injected with shIFT88-GFP-AAV (in left SN) and GFP-AAV (in right SN). Brains were collected 3 and 7 days after MPTP injections. The percentages of TUNEL-positive neurons in the SN with blockade of ciliogenesis were significantly higher at 7 days after MPTP injection compared to those at 3 days post-MPTP injections. Scale bars, 25 μm.
