# Supplementary figures and images for: Primary cilia mediate mitochondrial stress responses to promote dopamine neuron survival in a Parkinson’s disease model
Source: Cell Death Dis. 2019 Dec 16;10(12):952. doi: 10.1038/s41419-019-2184-y (PMC6915731; doi:10.1038/s41419-019-2184-y)

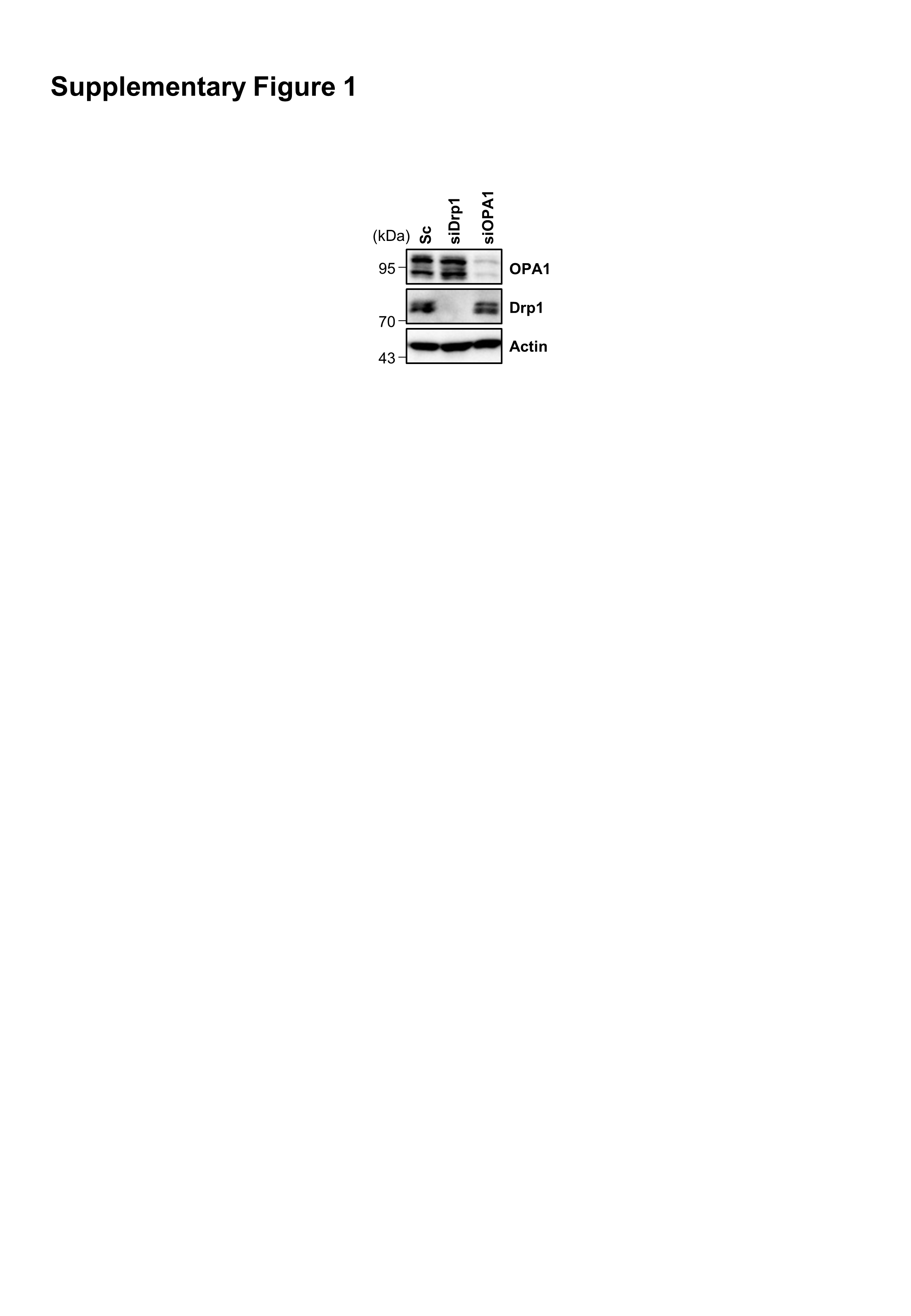

Supplement: Supplementary file 2 — Suppl. Fig. 1 [file 41419_2019_2184_MOESM2_ESM.tif]

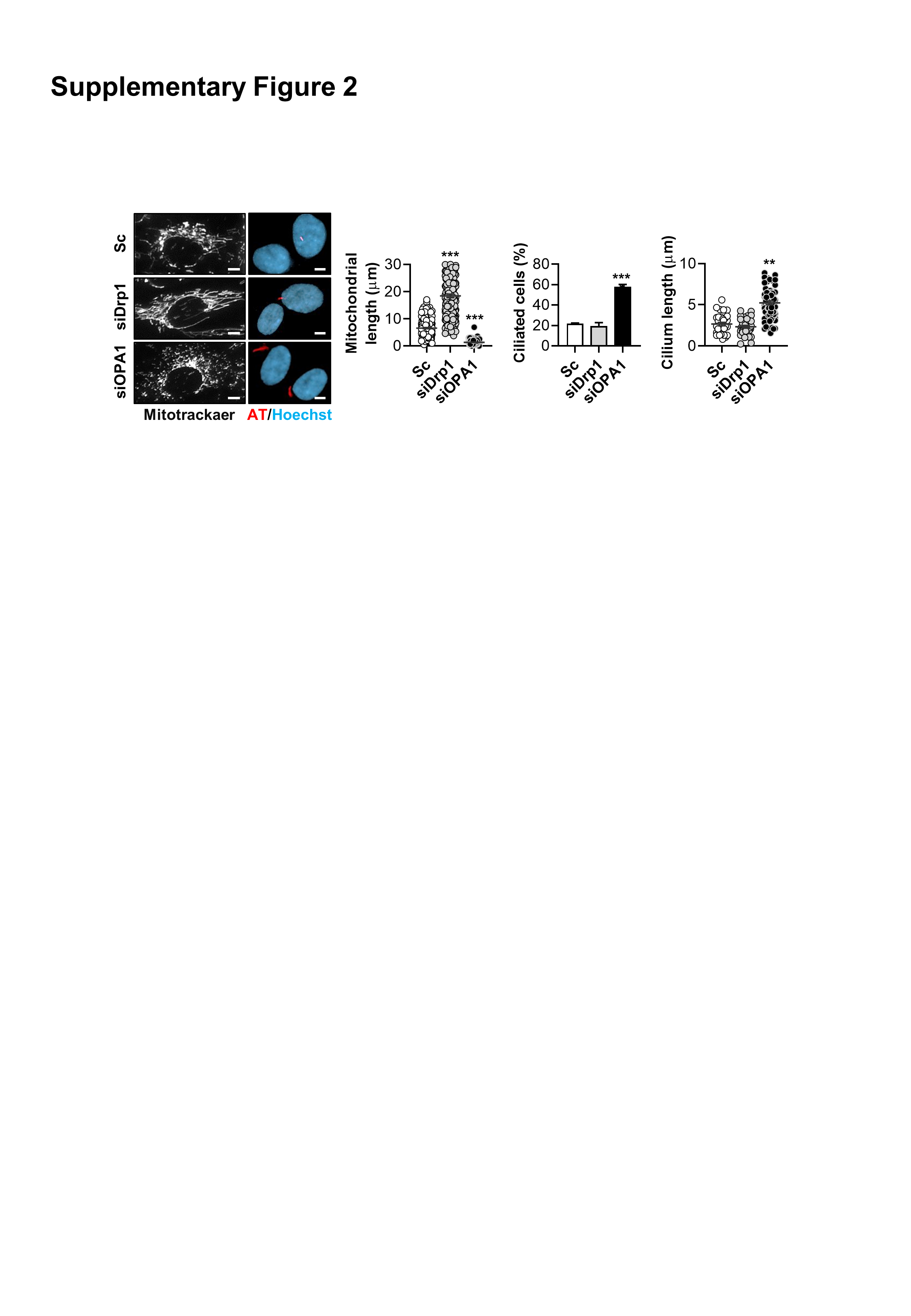

Supplement: Supplementary file 3 — Suppl. Fig. 2 [file 41419_2019_2184_MOESM3_ESM.tif]

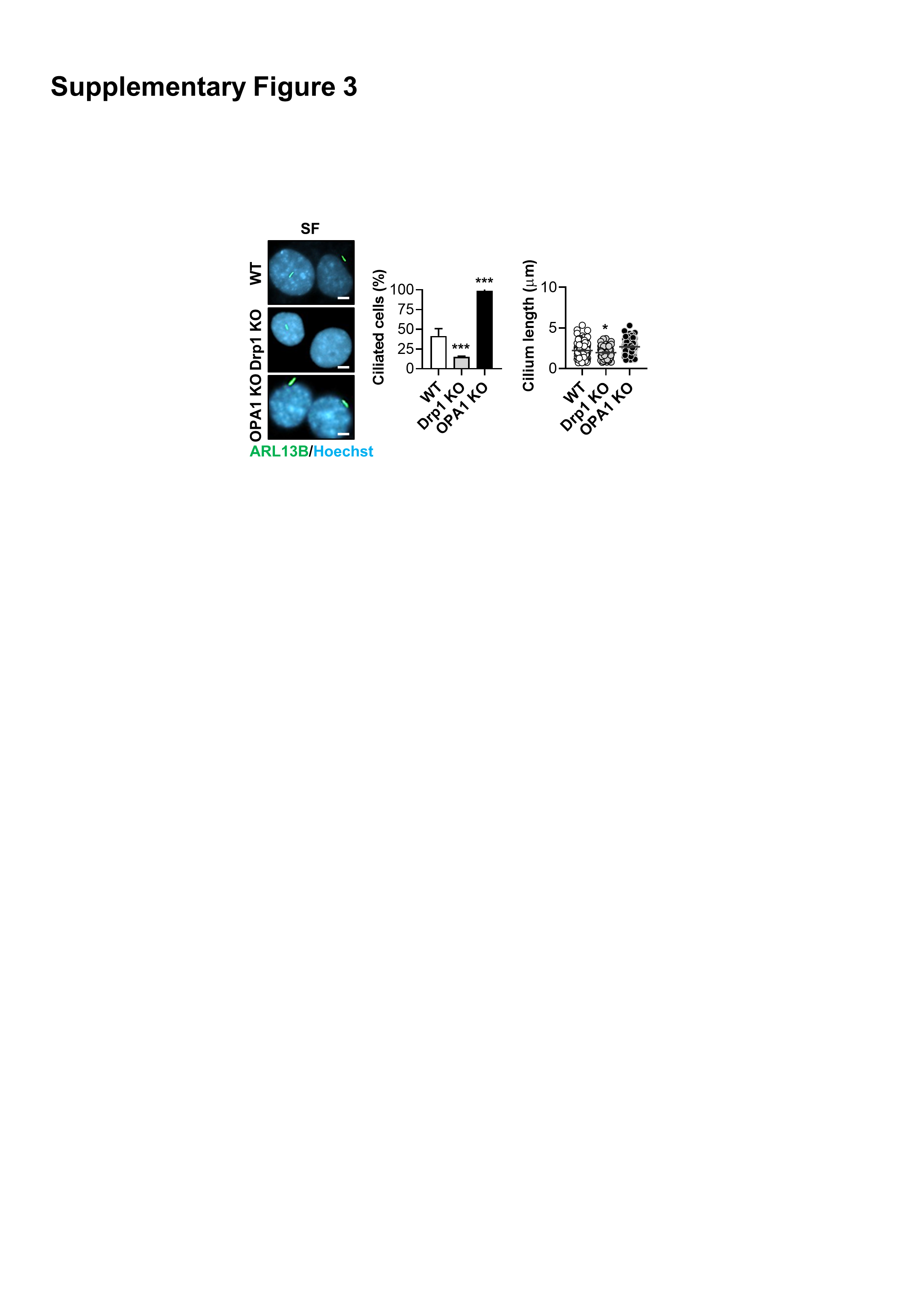

Supplement: Supplementary file 4 — Suppl. Fig. 3 [file 41419_2019_2184_MOESM4_ESM.tif]

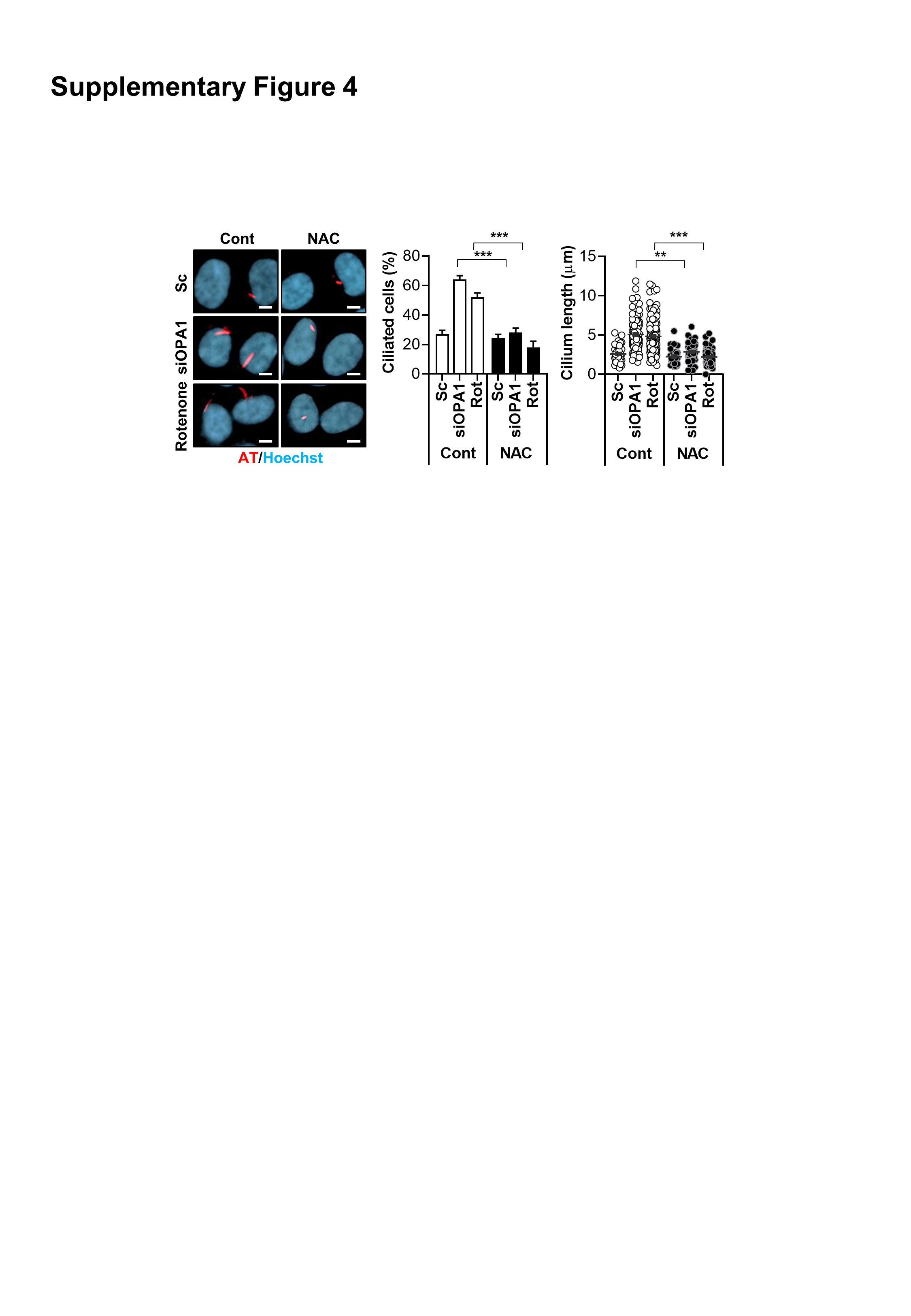

Supplement: Supplementary file 5 — Suppl. Fig. 4 [file 41419_2019_2184_MOESM5_ESM.tif]

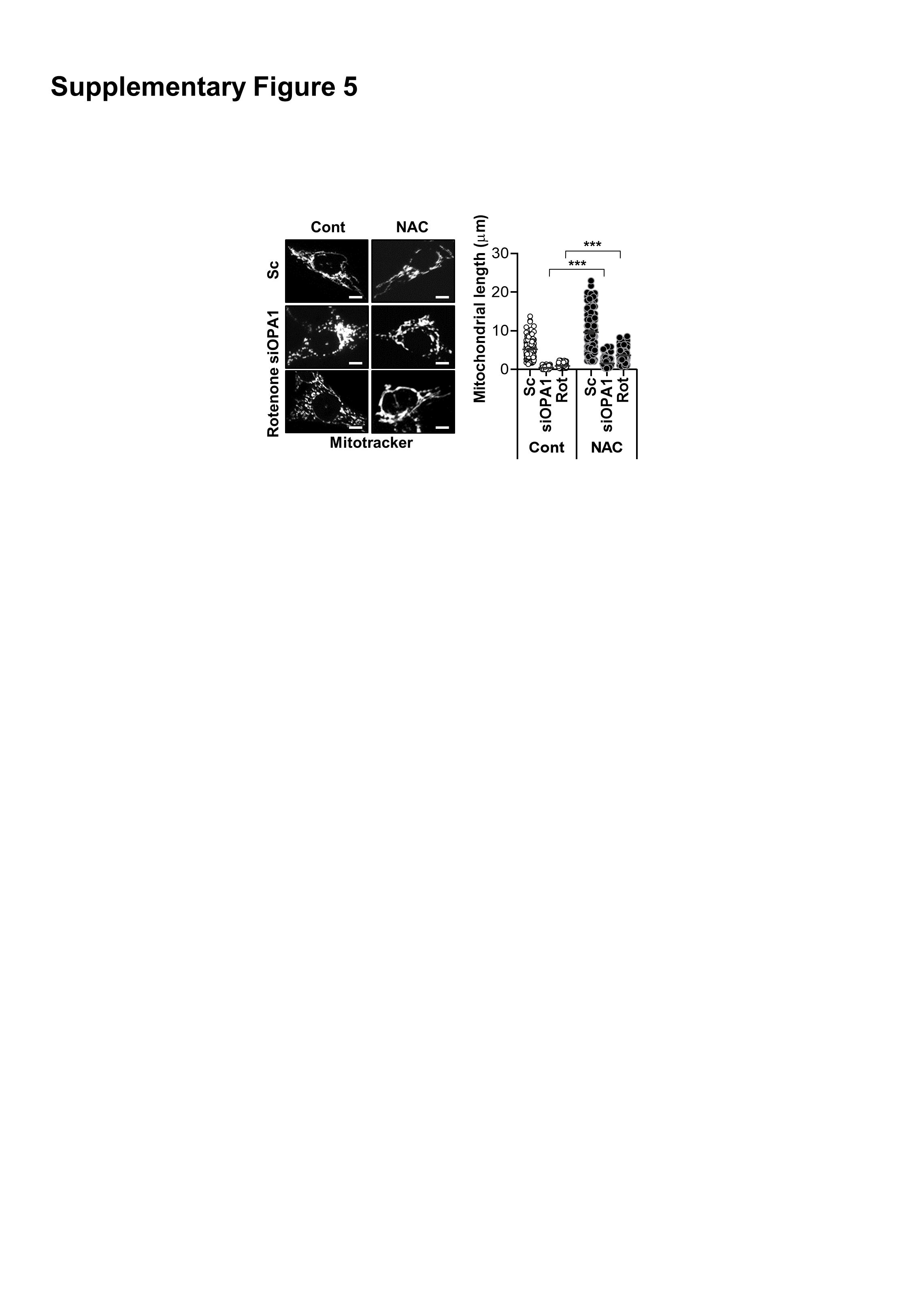

Supplement: Supplementary file 6 — Suppl. Fig. 5 [file 41419_2019_2184_MOESM6_ESM.tif]

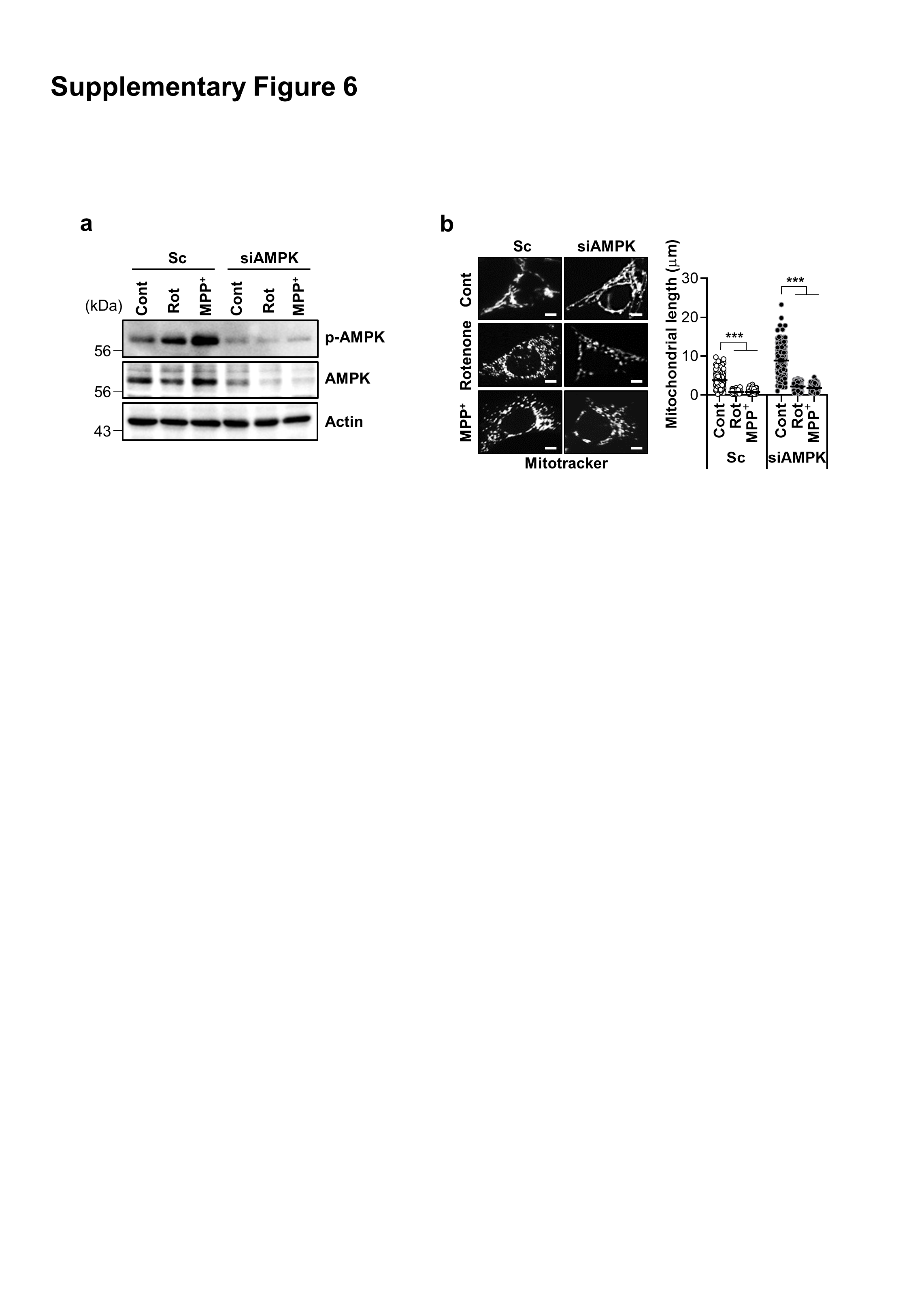

Supplement: Supplementary file 7 — Suppl. Fig. 6 [file 41419_2019_2184_MOESM7_ESM.tif]

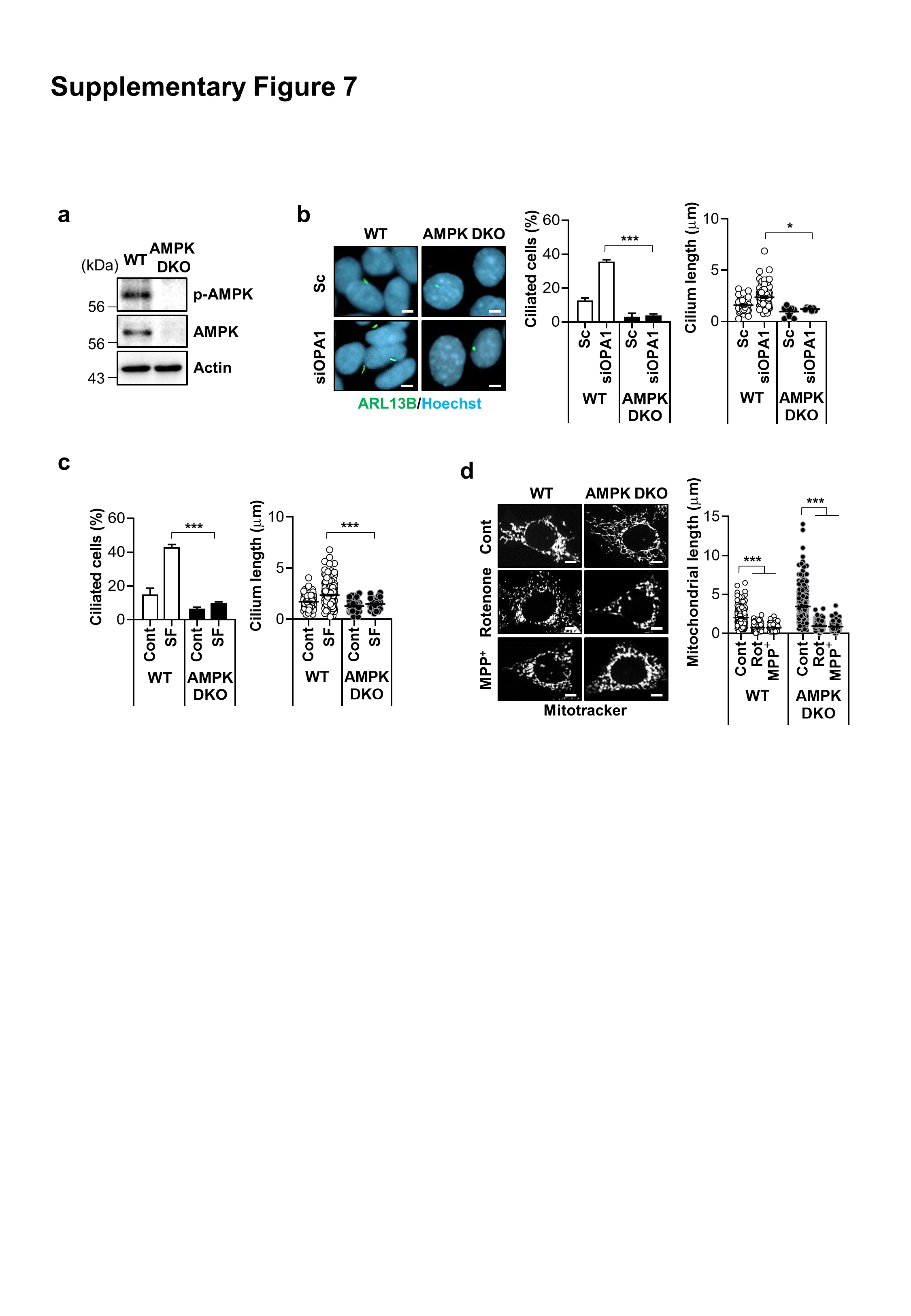

Supplement: Supplementary file 8 — Suppl. Fig. 7 [file 41419_2019_2184_MOESM8_ESM.tif]

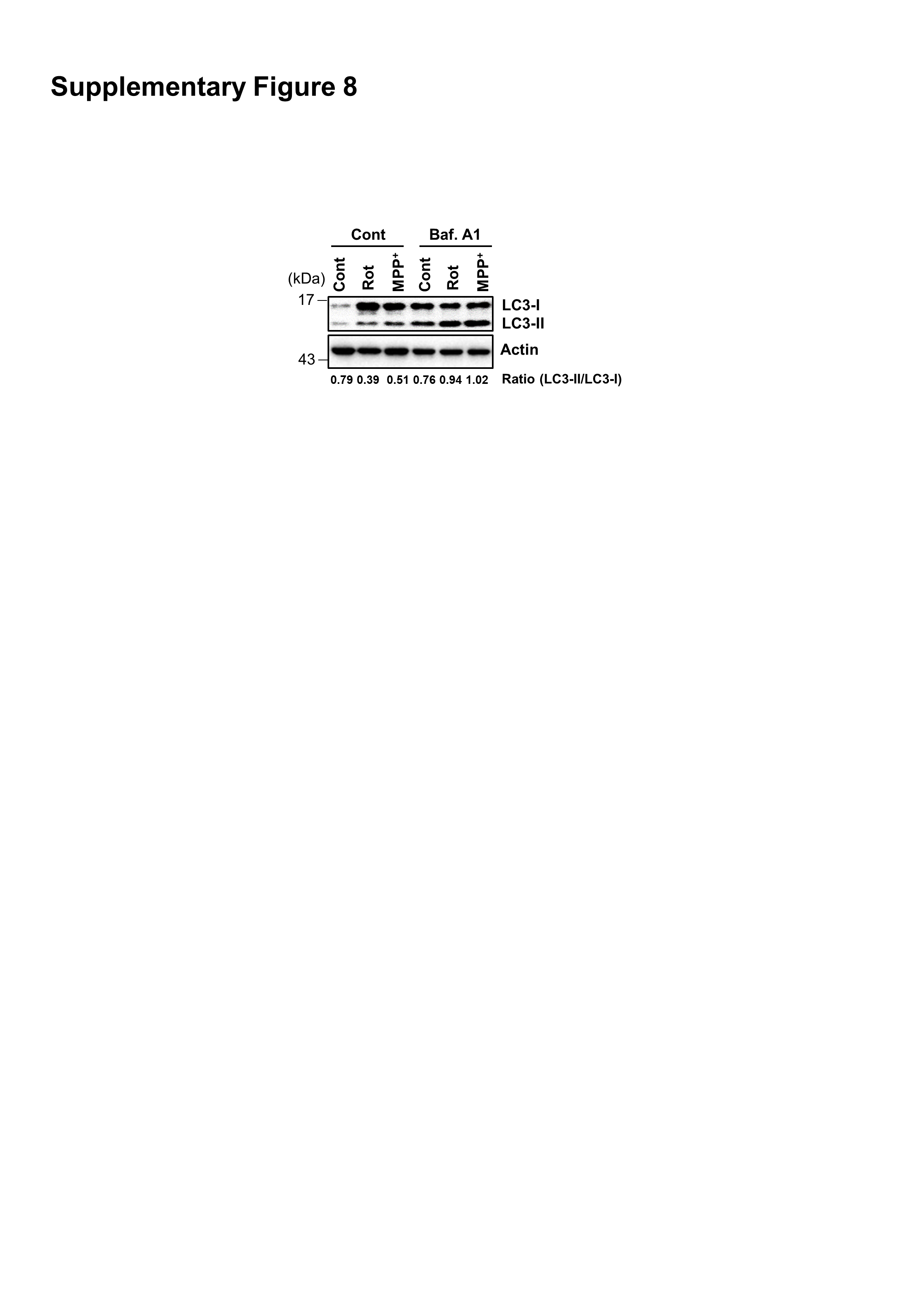

Supplement: Supplementary file 9 — Suppl. Fig. 8 [file 41419_2019_2184_MOESM9_ESM.tif]

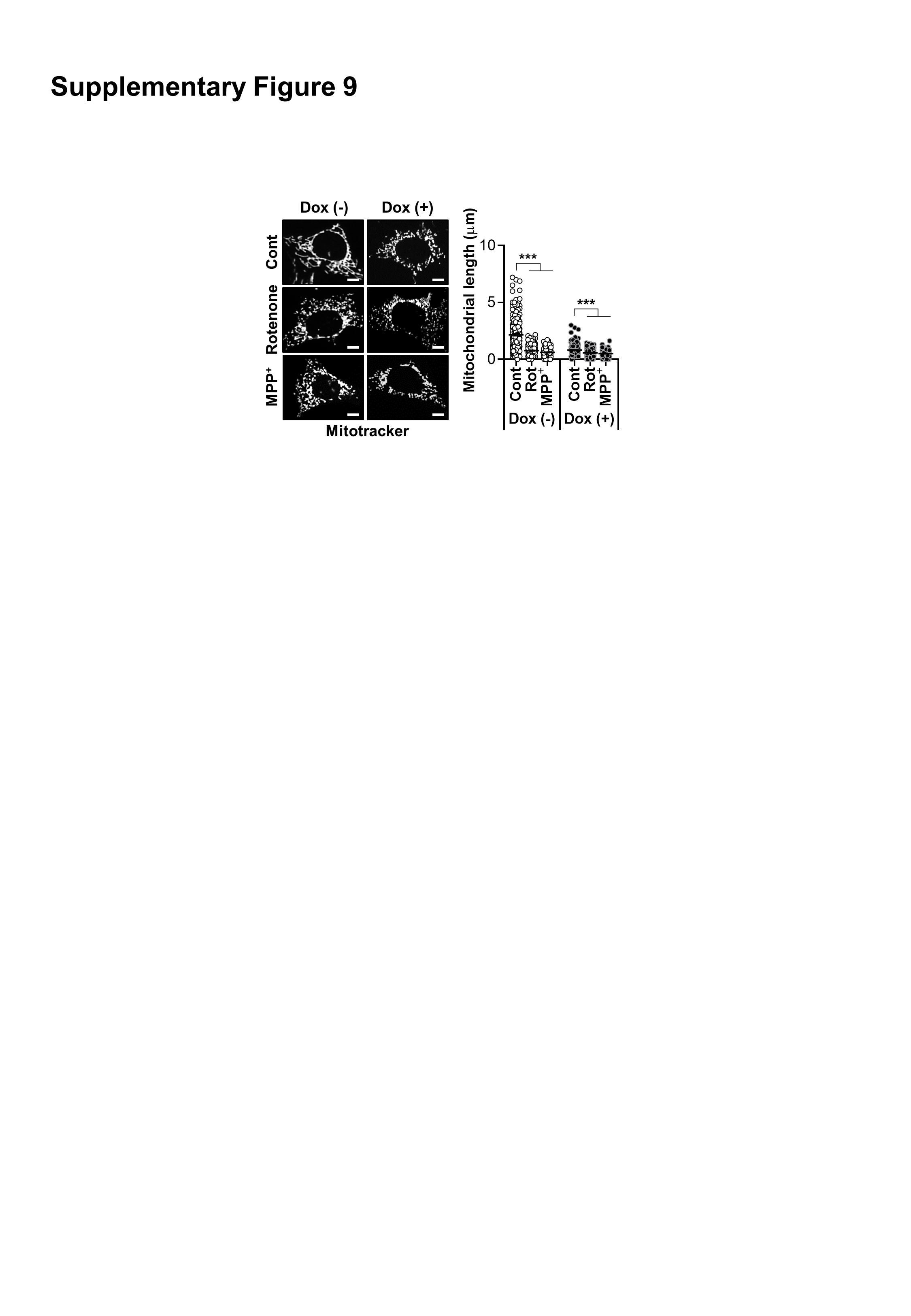

Supplement: Supplementary file 10 — Suppl. Fig. 9 [file 41419_2019_2184_MOESM10_ESM.tif]

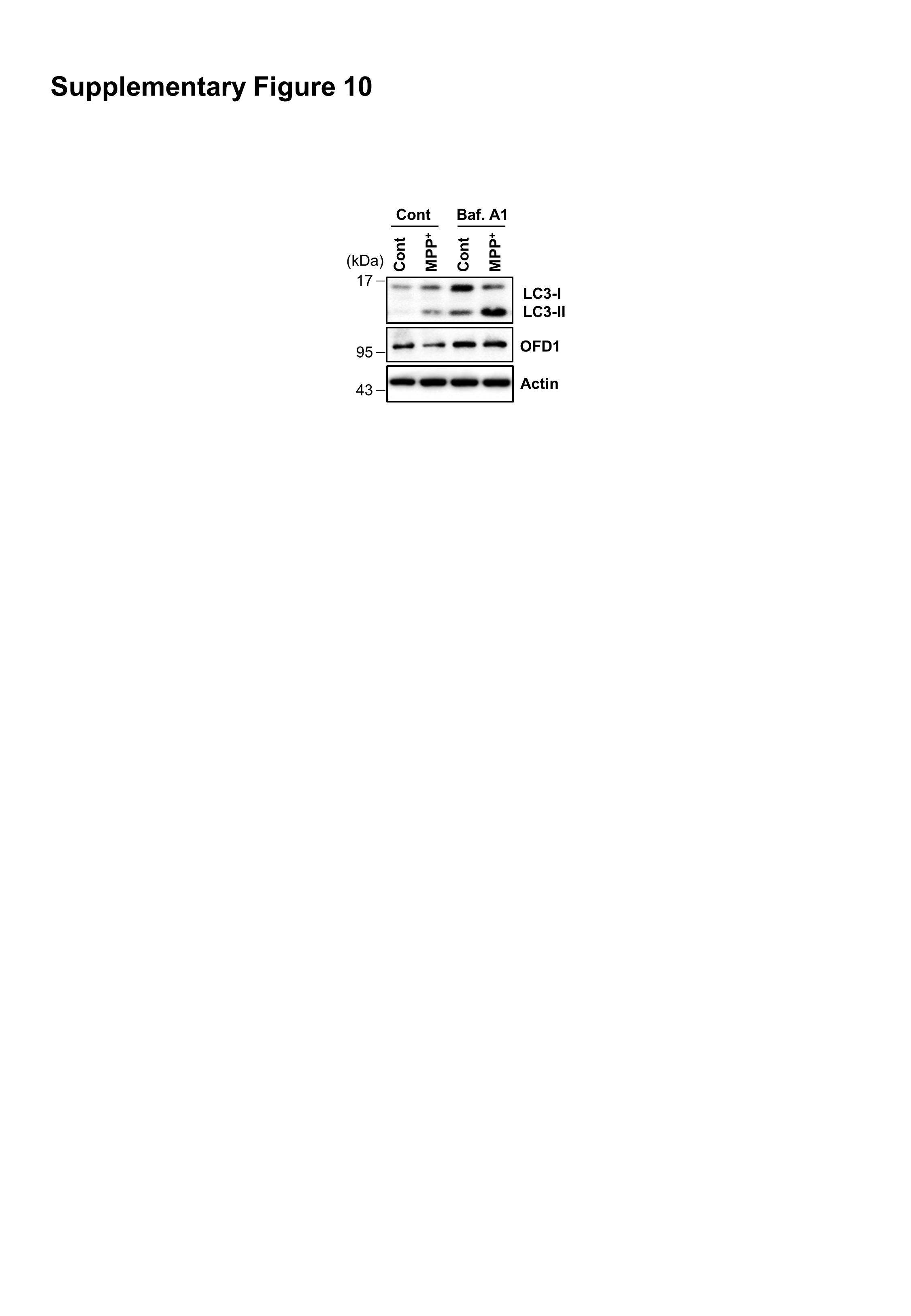

Supplement: Supplementary file 11 — Suppl. Fig. 10 [file 41419_2019_2184_MOESM11_ESM.tif]

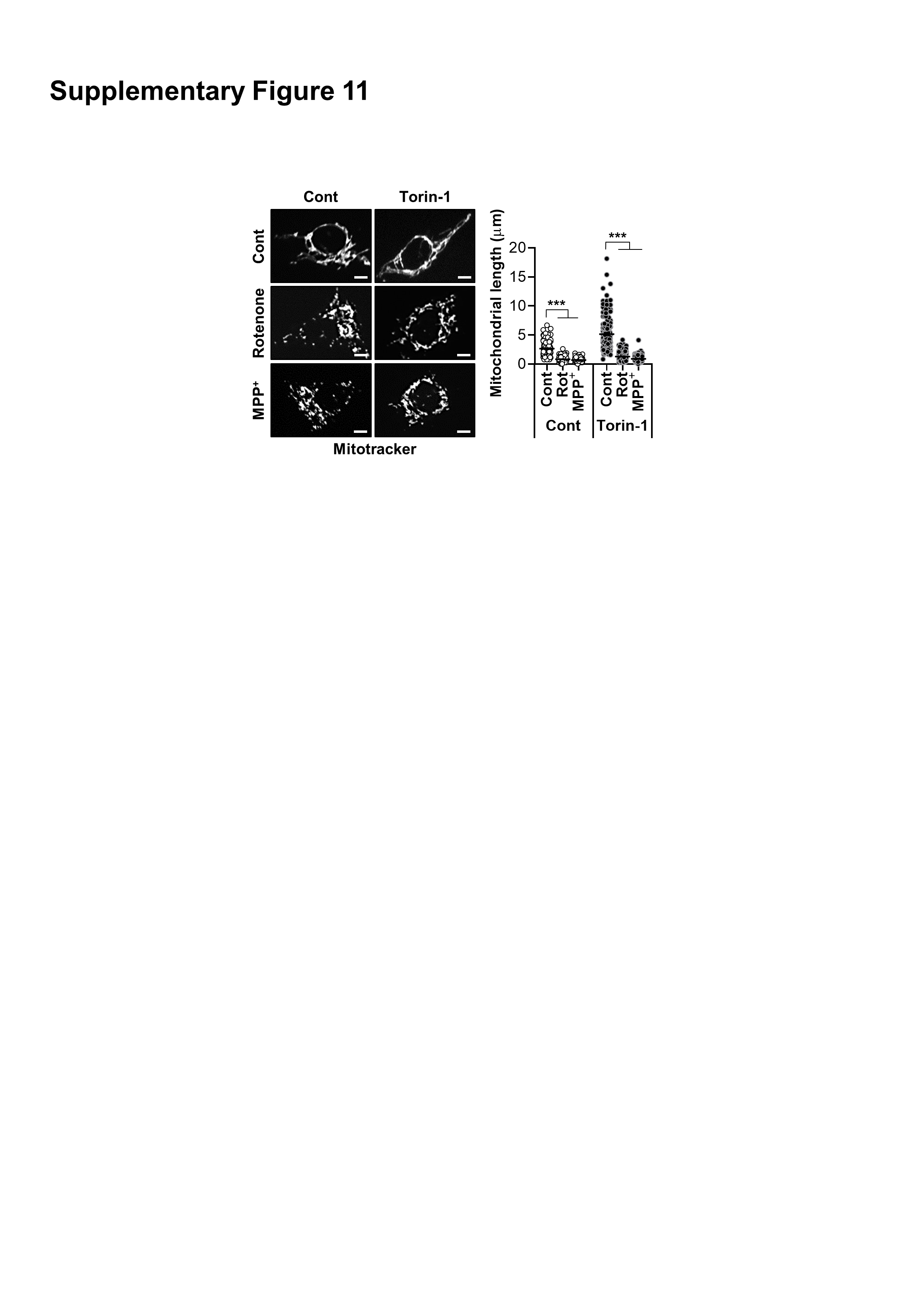

Supplement: Supplementary file 12 — Suppl. Fig. 11 [file 41419_2019_2184_MOESM12_ESM.tif]

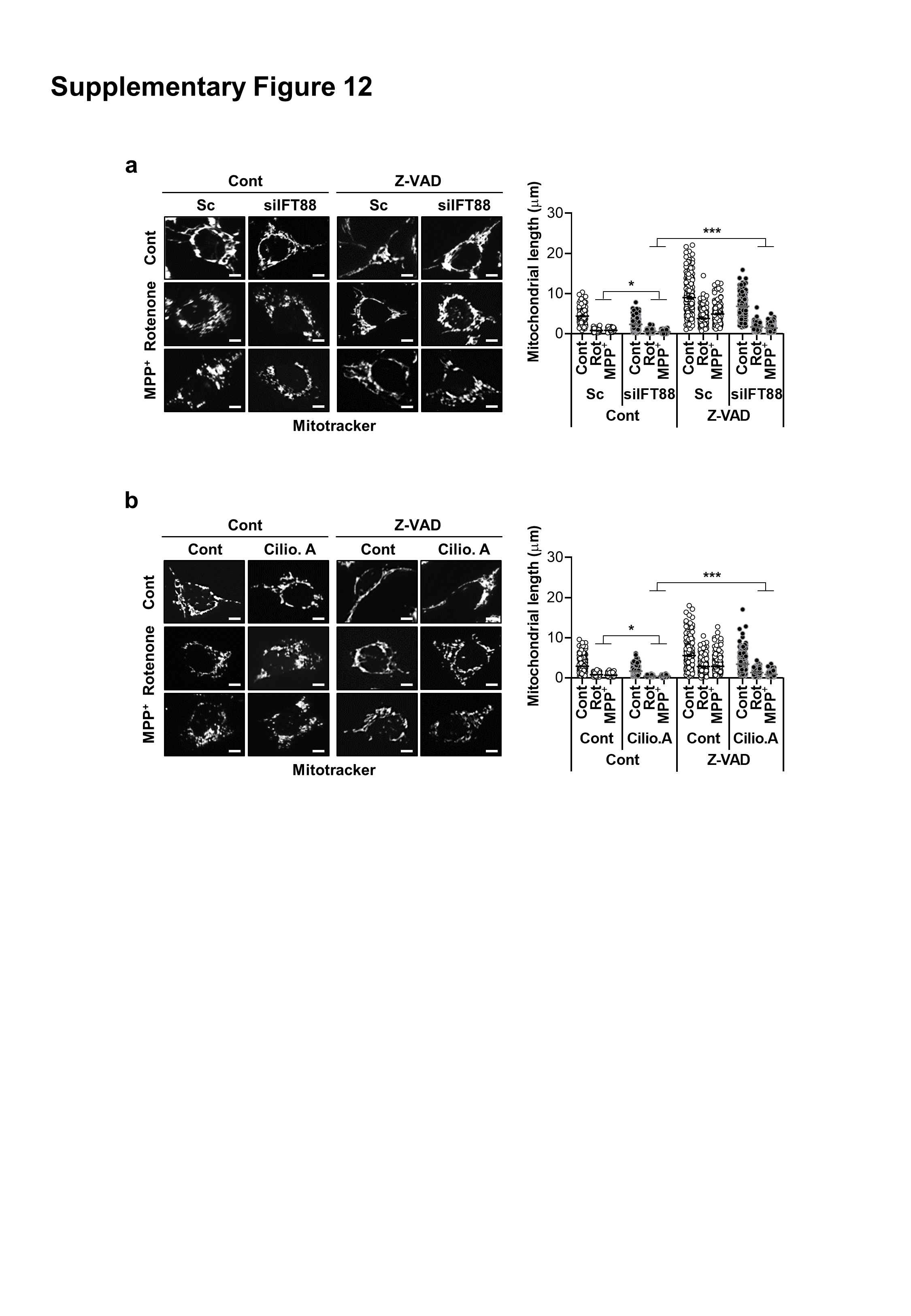

Supplement: Supplementary file 13 — Suppl. Fig. 12 [file 41419_2019_2184_MOESM13_ESM.tif]

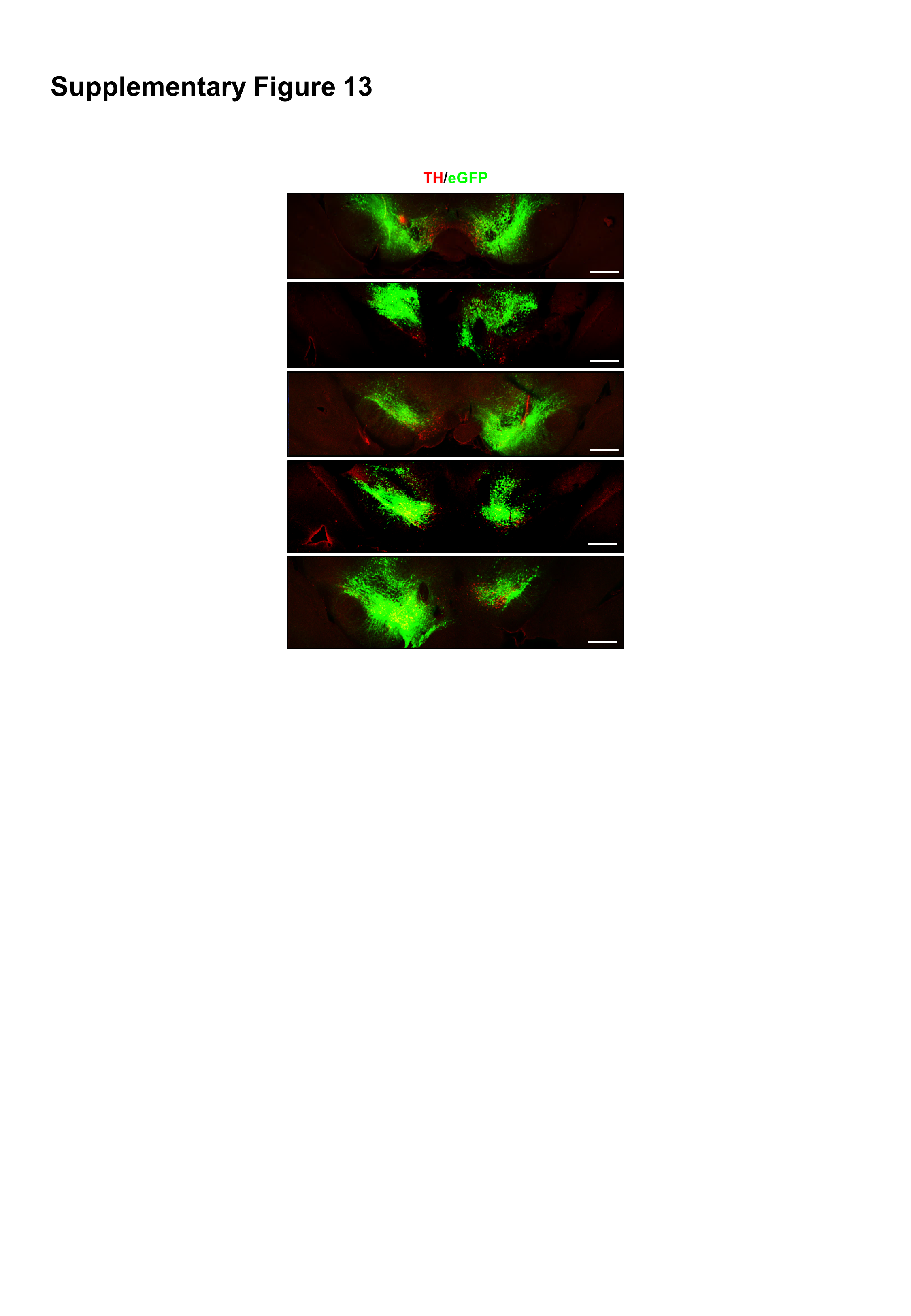

Supplement: Supplementary file 14 — Suppl. Fig. 13 [file 41419_2019_2184_MOESM14_ESM.tif]

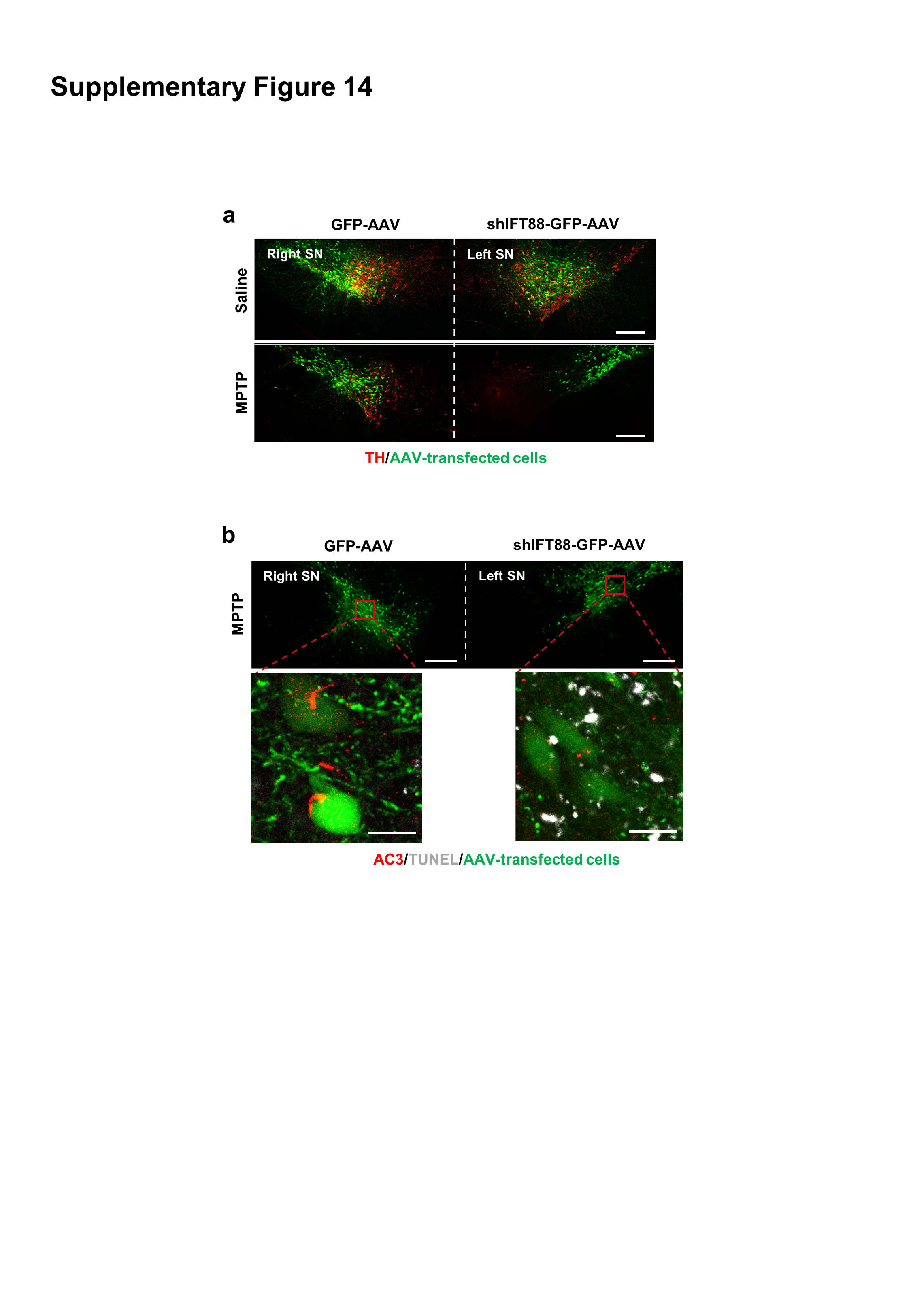

Supplement: Supplementary file 15 — Suppl. Fig. 14 [file 41419_2019_2184_MOESM15_ESM.tif]

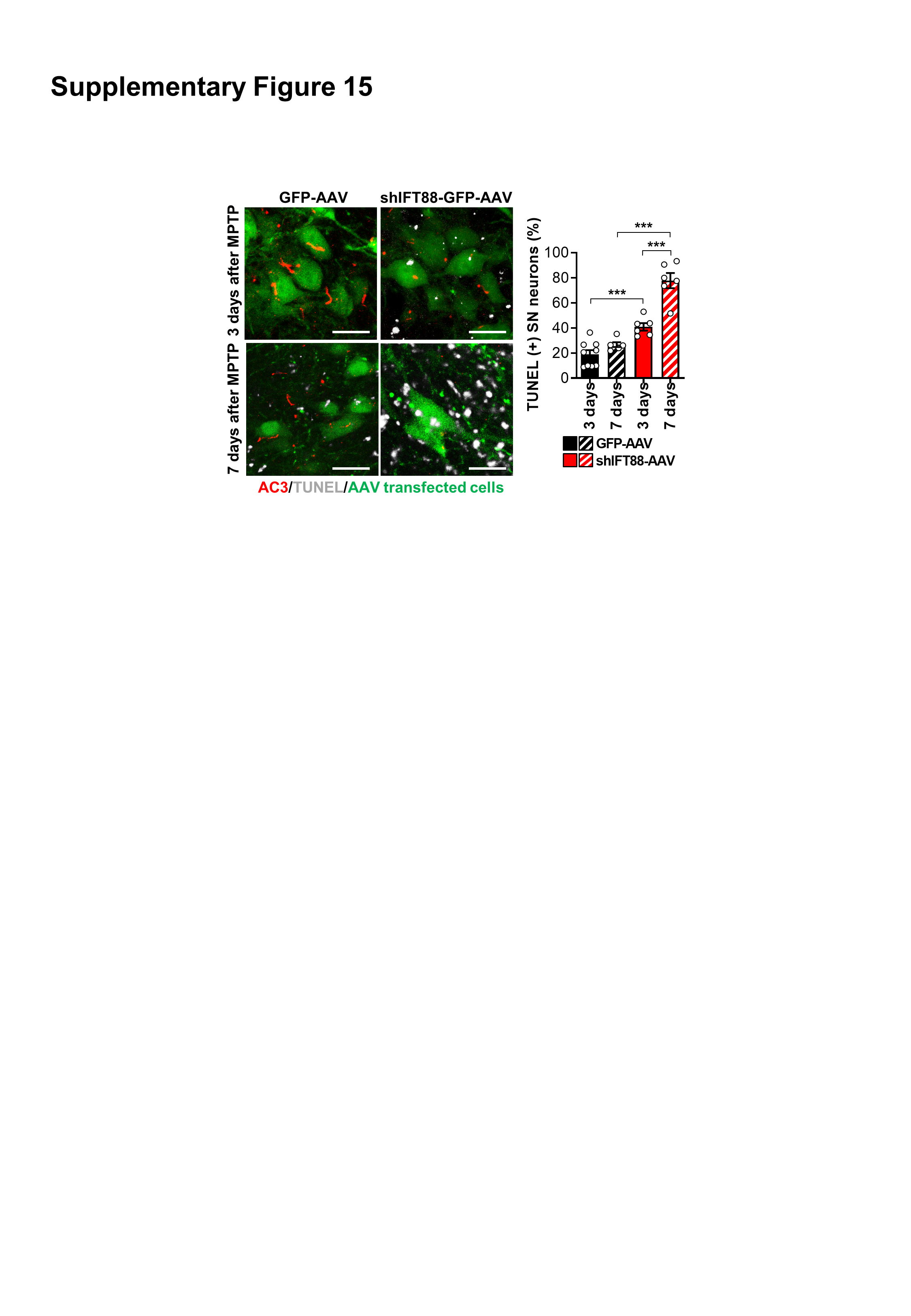

Supplement: Supplementary file 16 — Suppl. Fig. 15 [file 41419_2019_2184_MOESM16_ESM.tif]
